# Toll-like Receptor Signaling–deficient Cells Enhance Antitumor Activity of Cell-based Immunotherapy by Increasing Tumor Homing

**DOI:** 10.1158/2767-9764.CRC-22-0365

**Published:** 2023-03-01

**Authors:** Alvaro Morales-Molina, Miguel Ángel Rodriguez-Milla, Stefano Gambera, Teresa Cejalvo, Belén de Andrés, María-Luisa Gaspar, Javier García-Castro

**Affiliations:** 1 Cellular Biotechnology Unit, Instituto de Investigación de Enfermedades Raras, Instituto de Salud Carlos III (ISCIII), Madrid, Spain.; 2 Molecular Genetics of Angiogenesis Group, Centro Nacional de Investigaciones Cardiovasculares (CNIC), Madrid, Spain.; 3 Biological Products, Advanced Therapies and Biotechnology, Department of Medicines for Human Use, AEMPS, Madrid, Spain.; 4 Immunology Laboratory, Centro Nacional de Microbiología, Instituto de Salud Carlos III (ISCIII), Majadahonda, Spain.

## Abstract

Cancer immunotherapy aims to activate the immune system. Some immunotherapeutic agents can be loaded in carrier cells for delivering to the tumors. However, a challenge with cell-based therapies is the selection of the appropriate cells to produce effective clinical outcomes. We hypothesize that therapies based on cells presenting a natural low proinflammatory profile (“silent cells”) in the peripheral blood would result in better antitumor responses by increasing their homing to the tumor site. We studied our hypothesis in an immunotherapy model consisting of mesenchymal stromal cells (MSCs) carrying oncolytic adenoviruses for the treatment of immunocompetent mice. Toll-like receptor signaling–deficient cells (TLR4, TLR9, or MyD88 knockout) were used as “silent cells,” while regular MSCs were used as control. Although *in vitro* migration was similar in regular and knockout carrier cells, *in vivo* tumor homing of silent cells was significantly higher after systemic administration. This better homing to the tumor site was highly related to the mild immune response triggered by these silent cells in peripheral blood. As a result, the use of silent cells significantly improved the antitumor efficacy of the treatment in comparison with the use of regular MSCs. While cancer immunotherapies generally aim to boost local immune responses in the tumor microenvironment, low systemic inflammation after systemic administration of the treatment may indeed enhance their tumor homing and improve the overall antitumor effect. These findings highlight the importance of selecting appropriate donor cells as therapeutic carriers in cell-based therapies for cancer treatment.

**Significance:**

Cells carrying drugs, virus, or other antitumor agents are commonly used for the treatment of cancer. This research shows that silent cells are excellent carriers for immunotherapies, improving tumor homing and enhancing the antitumor effect.

## Introduction

Immunotherapies are currently used in a wide spectrum of cancers, including carcinomas (1), sarcomas (2), and hematologic malignancies (3). Viral immunotherapies are based on oncolytic viruses, which are natural or engineered viruses that selectively replicate in and kill cancer cells without harming the normal tissues. FDA and European Medicines Agency have already approved the first-in-class oncolytic immunotherapy talimogene laherparepvec (T-VEC; Imlygic) for the local treatment of unresectable melanoma (4).

Nowadays it is well accepted that antitumor effect of oncolytic viruses is not limited to the viral oncolysis, but also to the activation of the immune system triggered by the infection, subsequent signaling, and cell lysis (5), which reverses the immunosuppression state within tumors (6). Nevertheless, although immune responses to viral infections play a crucial role in activating local antitumor immune response, they also present some limitations to the systemic administration of naked oncolytic viruses. The physiologic barriers to overcome and the inability of the virus to home to the tumor site may reduce the intravenous delivery of viral particles reaching the malignancy (7). On the other hand, neutralization of the virus by the host immune system may occur, which leads to virus clearance and impedes the administration of multiple doses of treatment (5, 8).

As a solution, the use of different cell carriers presenting inherent tumor tropism has been considered for the systemic delivery of oncolytic viruses (7, 9). Immune cells, endothelial cells, progenitor cells such as mesenchymal stromal cells (MSCs) or neural stem cells, and even cancer cells have been investigated as vehicles in cell-based therapies, each of them presenting their advantages and disadvantages (7). Similarly, other delivery technologies for immunotherapies are under research to reduce off-target adverse effects (10).

A challenge in the development of successful cell-based therapies is the selection of the appropriate cell, as donor-dependent variations have been reported of high relevance (11, 12). Thus, election of suitable cells would be key to produce effective clinical outcomes. In this regard, inflammatory signaling may then act as a double-edged sword in cell therapies for cancer, especially depending on the route of administration. While local inflammation in the tumor microenvironment contributes to anticancer immunity, carrier cells showing proinflammatory profile may induce an immune response in the blood stream that compromises the delivery of the cells and/or the cargo. Understanding better the effect of inflammatory status of these cell vehicles may help to enhance cell-based therapies for cancer.

Here, we hypothesize that therapies based on cells presenting a natural low proinflammatory profile (“silent cells”) would result in better antitumor responses by increasing their homing to the tumor site. We tested our hypothesis using a cell-based virotherapy model consisting of MSCs carrying the oncolytic adenovirus (OAd) ICOVIR-5 (13). MSCs express different Toll-like receptors (TLR; ref. 14), a family of evolutionary conserved receptors that play crucial roles in the innate immune responses by recognizing pathogen-associated molecular patterns derived from various microbes (15). It has been proposed that priming of TLR4 leads to the polarization of MSCs into a proinflammatory MSC1 phenotype (16). For these reasons, we used MSCs from mice deficient for members of TLR signaling pathway as models of silent cells.

In this work, we studied the mechanism of action and antitumor efficacy of a cell-based virotherapy using MSCs knockout for TLR4^−/−^ (OAd-MSC TLR4^−/−^) and MSCs knockout for MyD88^−/−^ (OAd-MSC MyD88^−/−^) as “silent cells” in an immunocompetent mouse model of adenocarcinoma, in comparison with the use of MSCs wildtype (OAd-MSC WT).

Our results demonstrate that TLR pathway–deficient (silent) cells induce lower immune response in the blood stream and thus present higher homing to the tumor site than OAd-MSC WT. As a result, there is an increased local antitumor response that leads to higher tumor infiltration of immune cells and significant higher antitumor effect. These findings highlight the importance on selecting appropriate donor cells as therapeutic carriers and may be of relevance in cell-based therapies for cancer treatment.

## Materials and Methods

### Cell Culture and Adenoviral Infection

MSCs WT were obtained from abdominal adipose tissue of C57BL/6J and C57BL/10 mice, as described previously (17). MSCs TLR4^−/−^ were obtained from C57BL/10 TLR4^−/−^ mice and MSCs TLR9^−/−^ were obtained from C57BL/10 TLR9^−/−^ mice, while MSCs MyD88^−/−^ were obtained from C57BL/6 MyD88^−/−^ mice. Cell authentication was determined by fibroblast morphology of adherent cells and markers expression profile analysis by flow cytometry. The maximum passage number for these cells was 6. CMT64 cells are CMT64-6 clone, derived in our laboratory as previously described from parental cell line (18), a murine non–small cell lung carcinoma (RRID:CVCL_2406). CMT64-Luc cells were previously obtained in our laboratory (17). Cells were cultured in complete DMEM (Lonza Bioscience): DMEM supplemented with 10% FBS (Sigma-Aldrich), streptomycin (100 mg/mL), penicillin (100 U/mL), and glutamine (2 mmol/L; Lonza Bioscience); at 37°C in a humidified atmosphere with 5% CO_2_. *Mycoplasma* testing was routinely conducted using MycoAlert *Mycoplasma* Detection Kit (Lonza Bioscience).

For preparation of OAd-MSC, MSCs were infected with the OAd ICOVIR-5 (13) at a multiplicity of infection (MOI) of 200 for 2 hours at 37°C under suspension conditions in DMEM without FBS. Cells were washed with PBS to remove the virus from the cell culture supernatant.

### Toxicity Studies

For toxicity of MSCs after infection with OAd, 1 × 10^5^ cells per well were seeded in 6-well plates with complete DMEM. Apoptosis in MSCs was analyzed at 48 hours by flow cytometry using Annexin V apoptosis detection kit (BD Pharmingen).

Two *in vitro* transwell cocultures were performed to study the direct and indirect effect of MSCs and OAd-MSC on CMT64 cells. For direct coculture, 1 × 10^5^ CMT64 cells and 1 × 10^5^ MSCs/OAd-MSC per well were seeded in 6-well plates. 60 hours later, supernatant and cells were obtained, blocked, and labeled with anti-CD90 antibody (Miltenyi Biotec). For transwell coculture, 5 × 10^4^ CMT64-6 cells/well were seeded in 24-well plates and 5 × 10^4^ MSCs/OAd-MSC seeded in transwells (8 μm pore filters, BD Biosciences) coated with 0.1% gelatin (Sigma-Aldrich) for 72 hours. Apoptosis was quantified in CMT64 (CD90^−^) cells using Annexin V apoptosis detection kit (BD Pharmingen). CMT64 cells seeded alone were used as negative control, while CMT64 cells infected with ICOVIR-5 (MOI 200) were used as positive lytic control.

### Western Blot Analysis

MSCs and OAd-MSC were seeded with complete DMEM for 24 hours. Total proteins were extracted with SDS sample buffer and 1:100 protease inhibitor cocktail (Sigma-Aldrich). Proteins were separated by electrophoresis, transferred to polyvinylidene difluoride membranes (Bio-Rad Laboratories) and blocked with 2% milk in TBS. Mouse monoclonal c-Jun (clone 3/Jun; Abcam), Akt (pan; clone 40D4; Cell Signaling Technology), pJun (clone KM-1; Santa Cruz Biotechnology), rabbit monoclonal pAkt (Ser473, clone 2118; Abcam), and β-actin (clone AC-15; Sigma-Aldrich) were used as primary antibodies. Polyclonal goat anti-rabbit and anti-mouse immunoglobulins/HRP (Agilents Dako) were used as secondary antibodies for 1 hour at room temperature. Horseradish peroxidase (HRP) signal was detected with Immobilon Western Chemiluminiscent HRP Substrate (Merck Millipore).

### Cytokine Array

To study the secretome of MSCs and OAd-MSC, 4 × 10^4^ cells per well were seeded in 24-well plates with complete DMEM. After 24 hours, supernatants were collected and proinflammatory cytokines were analyzed using Proteome Profiler Mouse Array Panel A kit (R&D Systems). Cytokine expression was measured semiquantitatively by pixel density of duplicated spots using ImageJ software. Quantification of CXCL10 was confirmed using Mouse CRG-2/IP-10 Ray Bio ELISA Kit (RayBiotech). Differentially expressed proteins were further studied with the STRING (Search Tool for Recurring Instances of Neighbouring Genes) database to analyze the biological process in which they are involved. STRING is a tool that retrieves and displays all known and predicted associations between proteins, including both physical interactions as well as functional associations (19, 20).

To study the systemic proinflammatory status of mice treated with OAd-MSC, blood samples were acquired 48 hours after treatment administration and serum was obtained following standard protocol. Proinflammatory cytokines were analyzed and measured semiquantitatively as explained above.

### NFκB Activity in MSCs and Tumor Cells

A luciferase reporter system was used to evaluate the activation of NFκB pathway *in vitro* and *in vivo*. MSCs and CMT64 were transduced overnight with a lentiviral vector based on the pHAGE NFκB-TA-LUC-UBC-GFP-W plasmid (Addgene plasmid #49343) and NFκB-Luc MSCs and NFκB-Luc CMT64 cells were obtained.

To study the activation of NFκB in MSCs after adenoviral infection, cell lysis for total protein extraction was carried out 24 hours after infection and luciferase activity was assayed with the Luciferase Assay System (Promega Corporation).

To study the activation of NFκB in CMT64 tumor cells in coculture with OAd-MSC, 5 × 10^4^ NFκB-Luc CMT64 cells were seeded overnight in 24-well plates with complete DMEM. A total of 2 × 10^4^ OAd-MSC/well were seeded in the 24-well plates containing the NFκB-Luc CMT64 cells with DMEM (2% FBS). Luciferase activity was assayed at 24 and 48 hours with the Luciferase Assay System (Promega Corporation).

To study the activation of NFκB in the tumor, 1 × 10^6^ NFκB-Luc CMT64 cells were implanted subcutaneously in 7-week-old C57BL/6 mice. When the tumors were measurable, a single dose of PBS, OAd-MSC WT or OAd-MSC TLR4^−/−^ (1 × 10^5^ cells/mouse) was inoculated intraperitoneally. NFκB activation was monitored *in vivo* by luminescence imaging with IVIS 200 system (Caliper Life Sciences) at 24, 48, 72, and 96 hours after treatment administration. Data were analyzed using Living Image software (Xenogen).

### *In Vivo* Antitumor Efficacy

For *in vivo* antitumor experiments, 1 × 10^6^ CMT64 tumor cells were implanted subcutaneously in 7-week-old C57BL/6 mice. When the tumors were measurable, first doses of PBS or OAd-MSC (1 × 10^5^–10^6^ cells/mouse) were inoculated intraperitoneally. Administration of noninfected MSCs was also performed as control. A total of four doses separated by 4–5 days were administered. Tumor length (*L*), width (*W*), and height (*H*) were measured with a caliper periodically and tumor volume was calculated as (*L* × *W* × *H*)π/6. Area under the curve (AUC), a tool to measure kinetics of tumor growth in experimental animals (21), was computed using GraphPad Prism (GraphPad Software). Antitumor activity was calculated in relation to mean tumor volume of PBS group. Four weeks after the first treatment, mice were sacrificed and tumors were weighted and processed for flow cytometry or histology. The experiment was repeated three times.

Mice were maintained at the animal facility of the Instituto de Salud Carlos III. All animal experiments were approved by the Institutional Review Board of the ISCIII. The experimental protocols were also approved by the Consejería de Medio Ambiente of Comunidad de Madrid (PROEX 282.4/20 and PROEX 347/15).

### Adoptive Transfer of Splenocytes

Spleens from treated mice were mashed through a sterile 70 μm nylon mesh cell strainer, and red blood cells were lysed with Quicklysis buffer (Cytognos). A pool of splenocytes from treated mice (*n* = 5) was obtained. Subcutaneous CMT64 tumors were established into 7-week-old C57BL/6J mice as described above and randomly divided in three homogeneous groups (*n* = 5). Three days after tumor inoculation, 3 × 10^7^ splenocytes were transferred intravenously to each CMT64 tumor-bearing mouse. Tumor growth was followed for 28 days.

### Flow Cytometry

TLR4 was checked by flow cytometry using PE anti-mouse TLR4 (CD284)/MD2 complex antibody (clone MTS510; BioLegend). Expression of different membrane receptors was studied in OAd-MSC WT and TLR4^−/−^ by flow cytometry using the following antibodies: VCAM-1 (clone 553332, BD Pharmingen), TIM-3 (119715, BioLegend), MHC I (MA5-16562), MHC II (17-5321-81), and CD49b (12-5971) from eBioScience-Thermo Fisher Scientific; CXCR1 (FAB8628A-025), CCR1 (FAB5986P-025), and CXCR6 (FAB2145A) from R&D Systems; CXCR2 (130-115-635), CXCR3 (130-111-092), CXCR4 (130-102-223), CCR2 (130-108-722), and CCR9 (130-102-172) from Miltenyi Biotec.

To study tumor immune infiltrate, extracted tumors were digested with collagenase IV (1 mg/mL) in agitation for 40 minutes at 37°C and mechanically homogenized using a potter-elvehjem PTFE pestle when necessary. In the case of splenocytes, spleens were mechanically disaggregated. Cell suspensions were filtered through a sterile 70 μm nylon mesh cell strainer and red blood cells were lysed by incubation with Quicklysis buffer (Cytognos). Pools of cell suspensions were blocked with mouse FcR Blocking (Miltenyi Biotec) for 15 minutes and incubated with the following mouse mAbs for 20 minutes at 4°C: CD45 (30-F11), CD3 (145-2C11), CD4 (GK1.5), CD8 (53-6.7), CD11b (M1/70), CD11c (N418), CD206 (C068C2), MHCII (M5/114.15.2), Ly6C (AL-21), Ly6G (1A8-Ly6g), and CD49b (DX5), NK1.1 (PK136), all of them from eBioScience-Thermo Fisher Scientific; CD137 (1AH2PD-1) and PD-1 (29F.1A12) from BioLegend. Samples were acquired with MACSQuant Analyzer cytometer and analyzed using MACSQuantify analysis software (Miltenyi Biotec). Density of the following immune cell populations was normalized to tumor volume to allow for comparisons: leukocytes (CD45^+^); T cells (CD45^+^ CD3^+^), subclassified in Th cells (CD4^+^) and cytotoxic T cells (CD8^+^); natural killer (NK) cells (CD45^+^ CD11c^+^ CD49b^+^); myeloid cells (CD45^+^ CD11b^+^), subclassified in monocytes (Ly6G^−^ MHCII^−^), macrophages (Ly6G^−^ MHCII^+^), and neutrophils (Ly6G^+^ MHCII^−^). M1/M2 and N1/N2 subsets were also considered (CD206^−^/CD206^+^).

### Tumor Histology and IHC

Tumor samples were directly frozen, or fixed and embedded in paraffin. 5-μm-thick sections were stained with hematoxylin and eosin following standard protocol. IHC of infiltrating leukocytes was performed using BOND RXm (Leica Biosystems) with CD45 biotin anti-mouse antibody (catalog # 13-0451-85), from eBioScience-Thermo Fisher Scientific. Representative maps of the tumors and detailed images were obtained using NanoZoomer-SQ Digital slide scanner (C13140-01; Hamamatsu Photonics K.K.) and NDP.view2 viewing software (U12388-01, Hamamatsu Photonics K.K.).

### *In Vitro* Migration

24-well plate transwells (8 μm pore filters, BD Biosciences) were coated with 0.1% gelatin (Sigma-Aldrich) and 5 × 10^4^ MSCs/OAd-MSC were seeded in this upper chamber. As stimuli, 1 × 10^5^ CMT64 cells per well were seeded in the bottom chamber. DMEM alone was used as negative stimuli. After 24 hours, nonmigrated MSCs/OAd-MSC were removed and migrated cells were fixed with 10% formalin and stained with crystal violet. Cells from high‐power fields (HPF; 100X) were counted for each condition.

*In vitro* migration of OAd-MSC toward CMT64 tumor cells was also studied in a wound healing assay using the ibidi Culture-Insert 2 Well. A total of 35,000 OAd-MSC were seeded in one well, while 75,000 CMT64 cells were seeded as stimuli in the other well. After 24 hours, the insert was removed and cells were monitored for 24 hours after the gap creation. Individual cells were tracked with the Manual Tracking plugin for ImageJ and data were analyzed with the Chemotaxis and Migration Tool 2.0 (ibidi, free download from http://www.ibidi.de/applications/ap_chemo.html).

### Analysis of Blood Samples and Tumor Homing

Subcutaneous tumors were established in 7-week-old C57BL/6J mice by administration of 1 × 10^6^ CMT64 cells transduced with a lentiviral vector containing a firefly luciferase cassette (referred to as CMT64-Luc cells). MSCs/OAd-MSC were labeled with 8.33 mg/mL DIR buffer for 30 minutes at 37°C according to manufacturer (Caliper Life Sciences) and 1 × 10^5^ DIR^+^ MSCs/OAd-MSC per mouse were intraperitoneally injected. Nonlabeled (DIR^−^) OAd-MSC were also administered as negative control. A total of 48 hours after treatment administration, blood samples (200 μL) were obtained in BD Vacutainer Plus Blood Collection tube and centrifuged for 15 minutes at 2,000 × *g* to obtain serum. Complete blood count and biochemistry analysis were obtained using FUJI-DRI CHEM 4000i (FUJIFILM Corporation). The cytokine array analysis was performed as previously described using a pool of serum from the different groups.

*In vivo* tracking of DIR-labeled MSCs/OAd-MSC was studied by fluorescent imaging. Mice were sacrificed and tumors and organs were harvested 48 hours after treatment administration. Homing of MSCs/OAd-MSC to the tumor, lungs, spleen, liver, and kidney was monitored *ex vivo* by fluorescent imaging analysis of DIR signal with IVIS 200 system (Caliper Life Sciences). Data were analyzed using Living Image software (Xenogen). The experiment was repeated three times.

### Statistical Analysis

Data were analyzed and graphed with GraphPad Prism (GraphPad Software). *In vitro* results were expressed as mean + SD and *in vivo* results were expressed as mean + SEM. Significant differences between two groups were determined using parametric (unpaired *t* test) or nonparametric (Mann–Whitney) tests according to the normality of the data (Shapiro–Wilk test). For comparison of multiple groups, ANOVA followed by Tukey multiple comparisons tests (parametric), or Kruskall–Wallis test followed by Dunn multiple comparisons tests (nonparametric), were used according to the normality of the data. *, *P* < 0.05; **, *P* < 0.01; and ***, *P* < 0.001 were deemed statistically significant.

### Data Availability

The data generated in this study are available upon request from the corresponding author.

## Results

### OAd-MSC TLR4^−/−^ as a Model of Silent Cells

**FIGURE 1:**
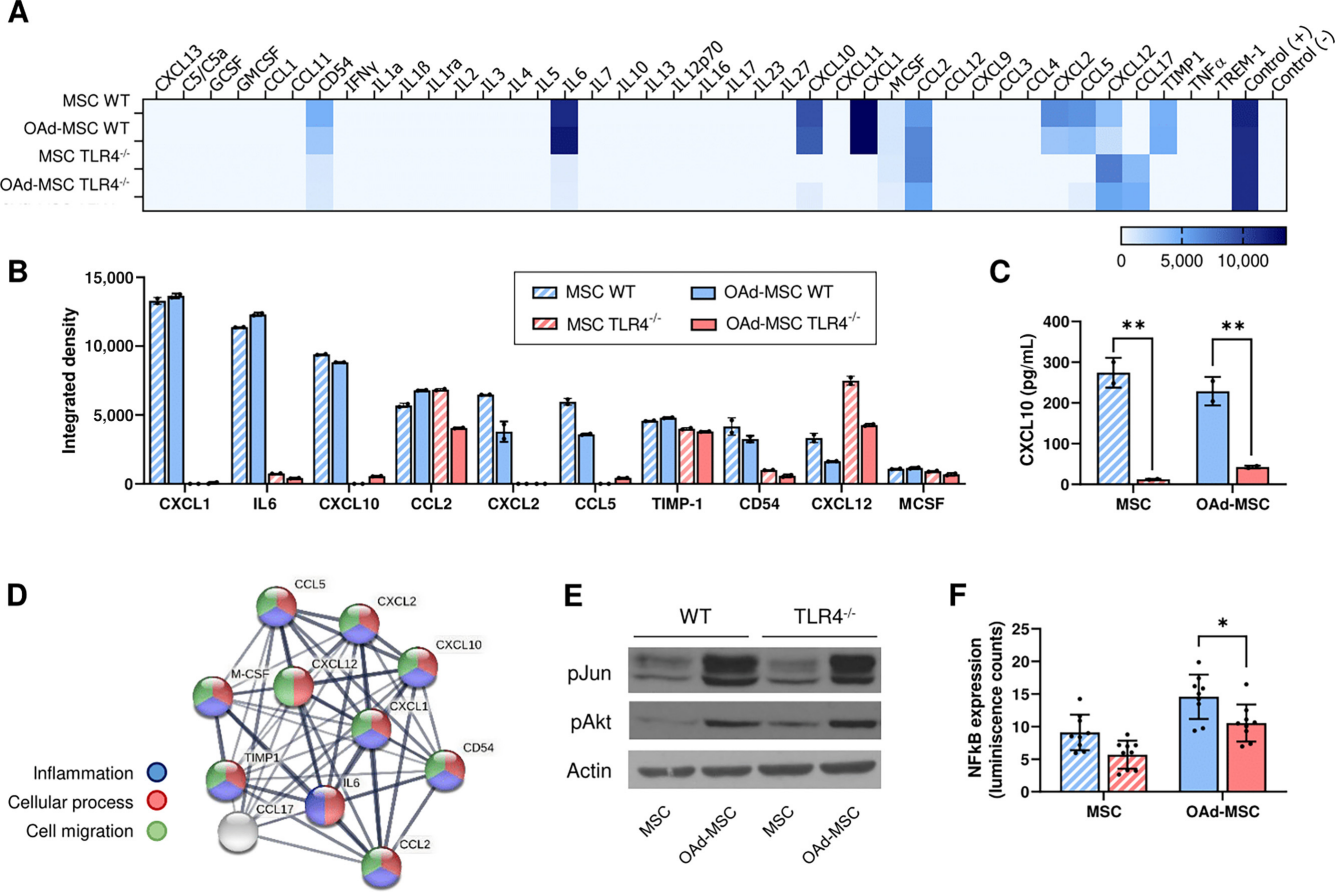
OAd-MSC TLR4^−/−^ as a model of silent cells. **A,** Heatmap showing the *in vitro* inflammatory secretome at 24 hours after infection with OAd. Mock-infected MSCs were used as control. Light blue represents the lowest expression while dark blue represents the highest expression. **B,** Quantification by integrated density of proinflammatory cytokines differentially secreted by OAd-MSC WT and OAd-MSC TLR4^−/−^. **C,** Quantification of secreted CXCL10 by ELISA. Two-way ANOVA followed by Tukey multiple comparisons test. **, *P* < 0.01. **D,** Protein–protein interaction map of differentially expressed cytokines generated by STRING protein network generator. Thickness of the lines represents the strength of the association between proteins. Colors of the nodes indicate the processes in which the proteins are involved: inflammation (blue), cellular process (red), or cell migration (green). **E,** Protein expression of phospho-Jun (pJun), phospho-Akt (pAkt), and Actin analyzed by Western blot analysis at 24 hours. **F,** Graph represents the NFκB activation at 24 hours expressed by luminescence. Two-way ANOVA followed by Tukey multiple comparisons test. **, *P* < 0.01. For the whole figure, stripped bars correspond to mock-infected MSCs while solid bars correspond to OAd-MSC (WT cells in blue; TLR4**^−/−^** cells in red).

Priming of TLR4 leads to the polarization of MSCs into a proinflammatory phenotype (16), so we used MSCs TLR4^−/−^ to mimic a silent carrier cell in contrast to MSCs WT. Absence of TLR4 in these MSCs was confirmed by flow cytometry (Supplementary Fig. S1A). To validate the use of MSCs TLR4^−/−^ as a model of silent cells, we first performed an *in vitro* study of their intracellular signaling and cytokine secretion (Fig. 1). In accordance with our hypothesis, both noninfected MSCs TLR4^−/−^ and OAd-MSC TLR4^−/−^ showed lower secretion profile of inflammatory cytokines compared with MSCs WT and OAd-MSC WT (Fig. 1A and B). From the 40 cytokines studied, OAd-MSC TLR4^−/−^ secreted less CXCL1, IL6, CXCL10, CCL2, TIMP-1, CXCL2, CCL5, and CD54 than OAd-MSC WT (Fig. 1B). The quantification of CXCL10 by ELISA confirmed the differences observed in the array, showing significant decreased expression in MSCs and OAd-MSC TLR4^−/−^ (Fig. 1C). The STRING analysis (19, 20) showed that cytokines differentially expressed in OAd-MSC WT and OAd-MSC TLR4^−/−^ are involved in cellular processes, inflammatory response and cell migration (Fig. 1D). We also confirmed that the infection with the OAd ICOVIR-5 induced similar cytotoxicity in MSCs TLR4^−/−^ than in MSCs WT (Supplementary Fig. S1B).

**FIGURE 2:**
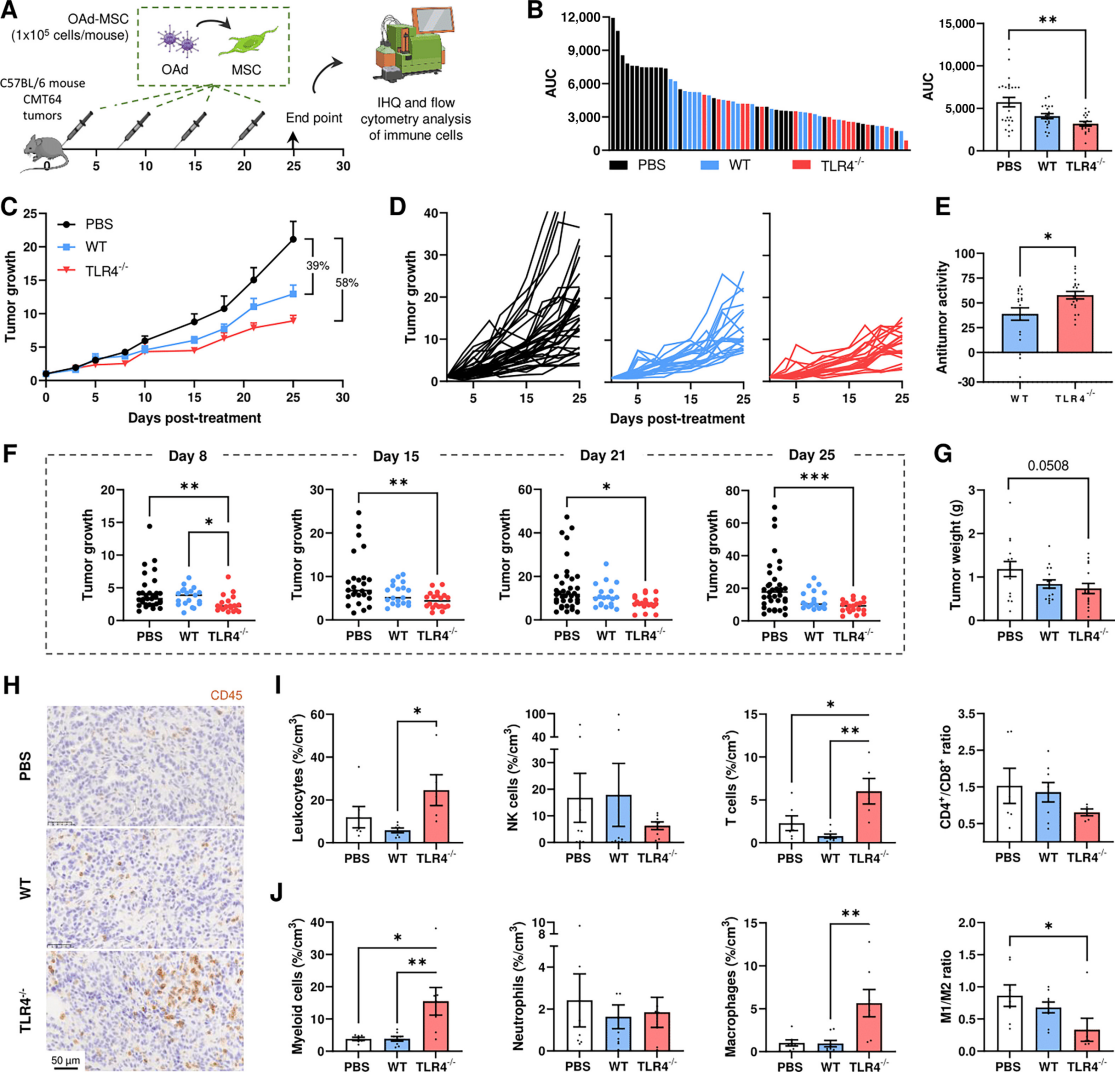
OAd-MSC TLR4^−/−^ improves the antitumor efficacy of OAd-MSC WT *in vivo*. **A,** Schematic illustration of *in vivo* experimental design. **B,** Graph on the left represents the individual AUC of mice treated with PBS (black), OAd-MSC WT (blue), or OAd-MSC TLR4^−/−^ (red). Graph on the right represents the AUC of treated groups expressed as mean + SEM (*n* = 17–25). Follow-up of tumor growth in mice treated with PBS (black, *n* = 35), OAd-MSC WT (blue, *n* = 20), or OAd-MSC TLR4^−/−^ (red, *n* = 20) represented as mean + SEM (**C**) and individual values (**D**). **E,** Antitumor activity of OAd-MSC WT and OAd-MSC TLR4^−/−^ in relation to control group (*n* = 20). Unpaired *t* test. **F,** Tumor growth of mice treated with PBS, OAd-MSC WT or OAd-MSC TLR4^−/−^ at indicated days. One-way ANOVA followed by Tukey multiple comparisons tests. **G,** Tumor weight of treated mice at endpoint (*n* = 15–16). One-way ANOVA followed by Tukey multiple comparisons tests. **H,** Representative images of IHC of CD45^+^ cells in treated tumors (*n* = 3–5). **I,** Density of tumor-infiltrating leukocytes, NK cells, and T cells at endpoint expressed as percentage per cm^3^ of tumor, as well as the CD4^+^/CD8^+^ ratio (*n* = 5–9). **J,** Density of tumor-infiltrating innate immune populations and ratio of proinflammatory/anti-inflammatory status of macrophages (M1/M2 ratio). One-way ANOVA followed by Tukey multiple comparisons test. *, *P* < 0.05; **, *P* < 0.01; ***, *P* < 0.001.

We have previously seen that infection with the OAd induces the expression of phospho(p)Jun and pAkt in MSCs (18, 22). Study of total and phosphorylated proteins showed similar levels in WT and TLR4^−/−^ cells after OAd infection (Fig. 1E; Supplementary Fig. S1C). Nevertheless, study of NFκB pathway by a luminescence reporter system showed that NFκB expression in OAd-MSC TLR4^−/−^ was significantly lower than in OAd-MSC WT (Fig. 1F).

Overall, these results show that OAd-MSC TLR4^−/−^ presents lower proinflammatory signaling than OAd-MSC WT, which validates its use as a model of silent carrier cells.

### OAd-MSC TLR4^−/−^ Improves the Antitumor Efficacy of OAd-MSC WT *In Vivo*

To study the antitumor efficacy of OAd-MSC TLR4^−/−^, we performed an *in vivo* experiment in C57BL/6 mice bearing CMT64 tumors, an immunocompetent mouse model previously developed by our group (17, 18). These mice were treated with repeated doses of OAd-MSC WT or OAd-MSC TLR4^−/−^ (1 × 10^6^ cells/mouse) every 5 days (Supplementary Fig. S2A).

While administration of OAd-MSC WT only induced an antitumor tendency, the administration of OAd-MSC TLR4^−/−^ resulted in significant antitumor effect after 10 days compared with control group (Supplementary Fig. S2B and S2C). Analysis of spleens from treated mice showed that both OAd-MSC WT and OAd-MSC TLR4^−/−^ induced changes in immune populations compared with PBS group (Supplementary Fig. S2D). The involvement of this activation of the immune response was further studied in a subsequent adoptive cell transfer of splenocytes from treated mice to new tumor-bearing mice (Supplementary Fig. S2A). Mice transferred with splenocytes from previous OAd-MSC WT and of OAd-MSC TLR4^−/−^ groups showed a trend toward decreased tumor growth compared to those treated with splenocytes from PBS group (Supplementary Fig. S2E), which confirmed the presence of antitumor immunity triggered by the cell-based virotherapy.

To better recapitulate the clinical conditions, we performed a new *in vivo* experiment at a dose similar to the human therapeutic setting (1 × 10^6^ cells/kg of weight), leading to the administration of 1 × 10^5^ cells/mouse every 5 days (Fig. 2A). Treatment with OAd-MSC TLR4^−/−^ induced significant antitumor effect (Fig. 2B and C). Furthermore, while treatment with OAd-MSC WT reduced tumor growth by 39%, this reduction was increased to 58% after treatment with OAd-MSC TLR4^−/−^ (Fig. 2C and D). Indeed, the antitumor activity of OAd-MSC TLR4^−/−^ is significantly higher than the observed using OAd-MSC WT (Fig. 2E–G). It is interesting to note that a general improvement of the antitumor effect was observed in all groups treated with this lower dose of OAd-MSC compared with the high dose.

These results indicate that silent cells (OAd-MSC TLR4^−/−^) improve the antitumor effect of OAd-MSC WT in our immunocompetent mouse model.

### Antitumor Effect is not Induced by MSCs TLR4^−/−^

Our previous work in immunocompetent models has demonstrated that this antitumor effect was not induced by the MSCs WT themselves (17), but this needed to be also confirmed in MSCs TLR4^−/−^. We then performed direct and indirect cocultures of MSCs and OAd-MSC with CMT64 tumor cells for 48 hours to avoid the oncolytic effect of the OAd, whose complete replication and release occurs at 72 hours, and therefore only study the influence of the MSC itself. Neither the secretome or cell-cell contact of WT nor TLR4^−/−^ cells induced any cytotoxic effect in the CMT64 cells *in vitro*, while direct infection with the OAd induced significant cell death (Supplementary Fig. S3A).

For further confirmation, we performed an *in vivo* experiment in our immunocompetent mice model (Supplementary Fig. S3B). After weekly administration of MSCs WT or MSCs TLR4^−/−^ (1 × 10^5^ cells/mouse), treated groups did not present any antitumor effect at endpoint (21 days after treatment) compared with control group (Supplementary Fig. S3C). In overall, these results indicate that MSCs TLR4^−/−^ do not induce any antitumor effect by themselves.

### OAd-MSC TLR4^−/−^ Induces High Tumor Infiltration of Innate and Adaptive Immune Cells

We then analyzed tumor-infiltrating immune cells by flow cytometry and IHC in mice treated with OAd-MSC. Interestingly, an increased density of leukocytes was observed in tumors treated with OAd-MSC TLR4^−/−^ compared with those treated with either PBS or OAd-MSC WT (Fig. 2H and I). Treatment with OAd-MSC TLR4^−/−^ also induced increased density of tumor-infiltrating T cells, as well as a lower CD4^+^/CD8^+^ ratio (Fig. 2I).

This increased tumor infiltration in the OAd-MSC TLR4^−/−^ group was also observed in regard to innate immune populations, as the group treated with OAd-MSC TLR4^−/−^ showed significant higher density of myeloid cells and macrophages than those treated with either PBS or OAd-MSC WT (Fig. 2J). Moreover, tumors treated with OAd-MSC TLR4^−/−^ also showed lower M1/M2 and N1/N2 ratios than those treated with PBS (Fig. 2J; Supplementary Fig. S4). To study the systemic status of the immune system, we also studied the immune populations in the spleen. Nevertheless, no clear differences in any immune population were observed between treated and nontreated groups (Supplementary Fig. S5).

These results indicate that silent cells (OAd-MSC TLR4^−/−^) induce high tumor infiltration of innate and adaptive immune cells.

### OAd-MSC WT and OAd-MSC TLR4^−/−^ Equally Migrate to Tumor Cells *In Vitro*

According to our hypothesis, the better antitumor effect of OAd-MSC TLR4^−/−^ could be related to a higher capability of migration to the malignancy, as indeed most of the cytokines differentially expressed in OAd-MSC WT and OAd-MSC TLR4^−/−^ were involved in this process (Fig. 1D).

**FIGURE 3:**
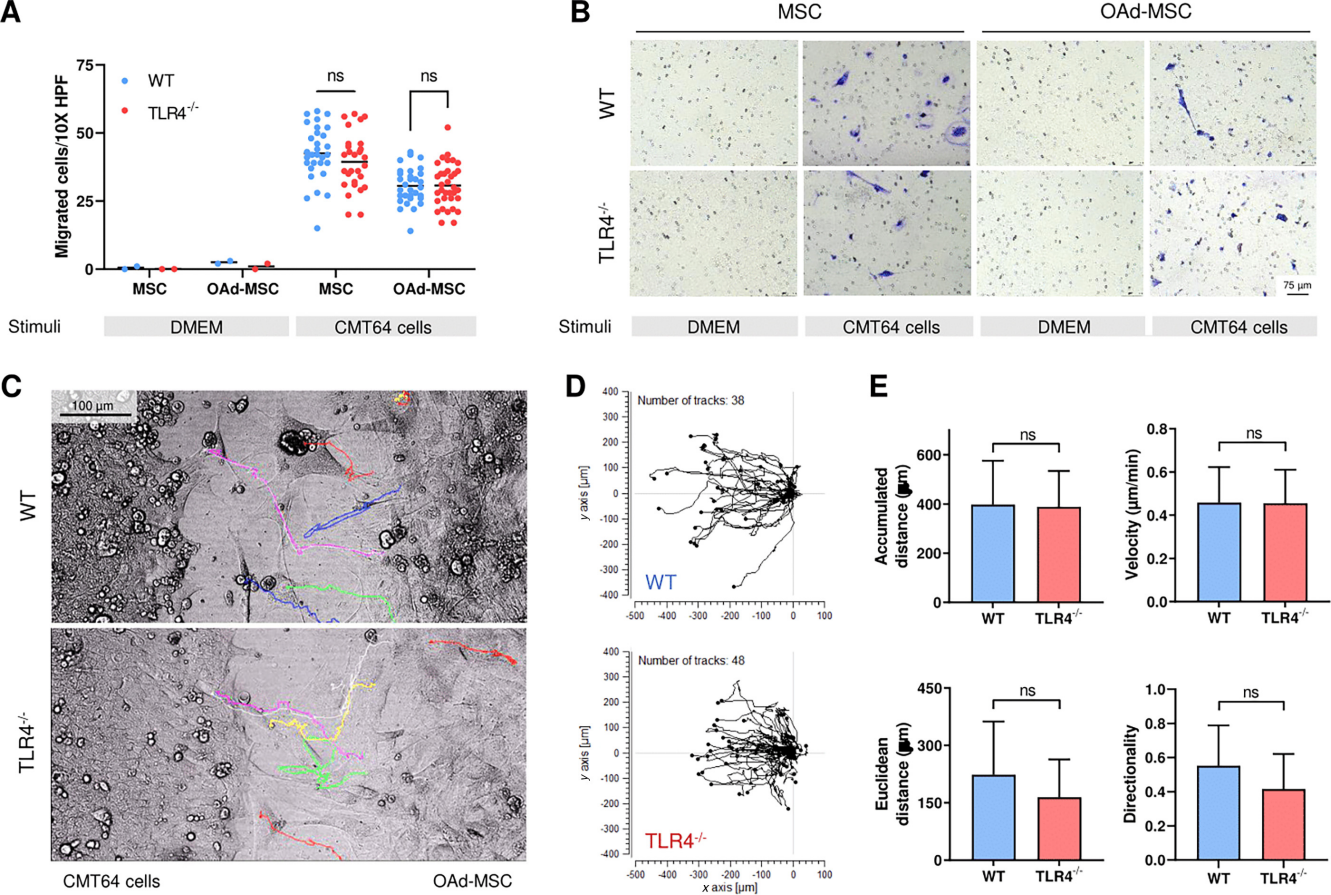
OAd-MSC WT and OAd-MSC TLR4^−/−^ equally migrates to tumor cells *in vitro*. **A,** Transwell migration assay of MSCs and OAd-MSC toward CMT64 tumor cells at 24 hours (*n* = 30). DMEM alone as stimuli was used as negative control (*n* = 2). Migrated cells were quantified in 10X HPF. Two-way followed by Tukey multiple comparisons test. ns, not significant; ***, *P* < 0.001. **B,** Representative images of migrated cells. **C,** Representative images of migrating OAd-MSC (right) seeded in 2-well inserts in the presence of CMT64 tumor cells (left). Color lines represent *in vivo* tracking of individual OAd-MSC for 24 hours. **D,** Trajectory plot of tracked OAd-MSC (*n* = 38 in OAd-MSC WT, *n* = 48 in OAd-MSC TLR4^−/−^). **E,** Parameters measured from tracked cell trajectories of migrating OAd-MSC toward tumor cells.

We first performed an *in vitro* transwell migration assay in which MSCs and OAd-MSC were exposed to medium (negative control) or to CMT64 cells seeded previously on the well as stimuli. A total of 24 hours later, migration of MSCs and OAd-MSC toward tumor cells was confirmed in both WT and TLR4^−/−^ cells, while no basal migration was observed in the absence of stimuli (Fig. 3A and B). No differences were observed between WT and TLR4^−/−^ cells (Fig. 3A).

To corroborate these results, we performed additional *in vitro* experiments using 2-well silicone inserts with a defined cell-free gap. OAd-MSC WT or OAd-MSC TLR4^−/−^ were then seeded in the presence of CMT64 tumor cells and *in vivo* tracking of individual cells was performed for 24 hours (Fig. 3C; Supplementary Video S1 and S2). Both OAd-MSC WT and OAd-MSC TLR4^−/−^ showed similar pattern of migration toward tumor cells (Fig. 3D), and no differences in accumulated distance, velocity, Euclidean distance or directionality were observed between them (Fig. 3E).

Infection of the different MSCs with the OAd could induce different expression of receptors, so we studied a panel of 13 cell surface receptors involved in migration and/or inflammation processes (Supplementary Fig. S6). In general, OAd-MSC WT and OAd-MSC TLR4^−/−^ showed similar pattern expression of cell membrane receptors *in vitro*.

As overall, these results indicate that OAd-MSC WT and OAd-MSC TLR4^−/−^ present similar capacity of *in vitro* migration.

### OAd-MSC TLR4^−/−^ Induces Lower Inflammatory Response in the Peripheral Blood After Systemic Administration than OAd-MSC WT

**FIGURE 4:**
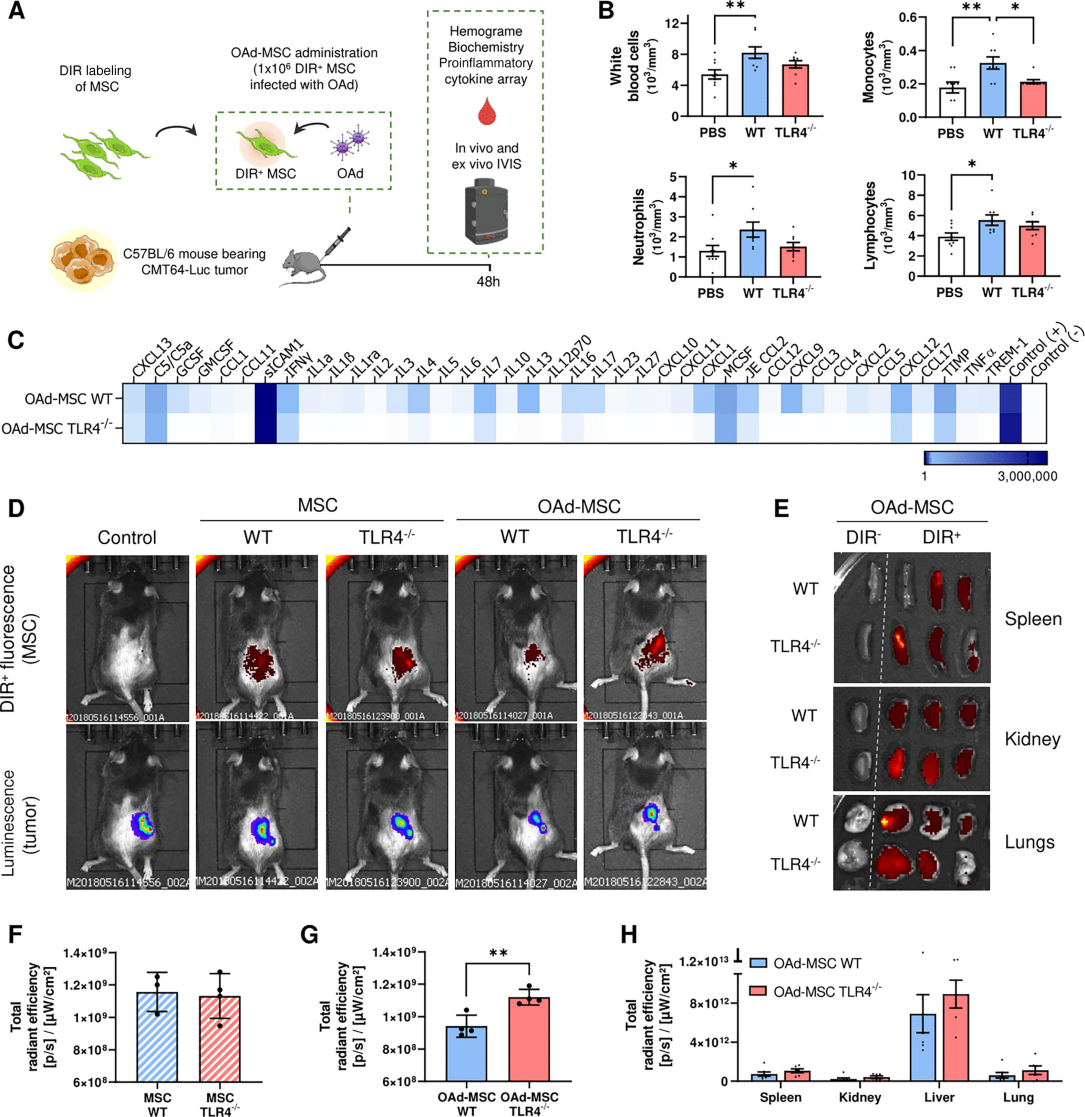
OAd-MSC TLR4^−/−^ present higher homing to the tumor site than OAd-MSC WT *in vivo*. **A,** Schematic illustration of *in vivo* experimental design. **B,** Immune populations from complete blood count (*n* = 8) showing differences at 48 hours after administration of PBS (white), OAd-MSC WT (blue), and OAd-MSC TLR4^−/−^ (red). One-way ANOVA followed by Tukey multiple comparisons test. *, *P* < 0.05; **, *P* < 0.01. **C,** Heatmap showing inflammatory cytokines in serum at 48 hours after administration of OAd-MSC WT or OAd-MSC TLR4^−/−^ (pool from *n* = 4). White represents the lowest expression while dark blue represents the highest expression. **D,***In vivo* homing of MSCs and OAd-MSC to tumor site 48 hours after treatment administration. DIR^+^ fluorescence detects labeled MSCs, while bioluminescence detects CMT64-Luc tumors. Unlabeled OAd-MSC were used as negative control. All images are displayed at the same fluorescence scale. **E,***Ex vivo* homing of MSCs and OAd-MSC to different organs extracted at 48 hours. Samples on the first column correspond to negative controls. *Ex vivo* quantification of fluorescence (DIR^+^) signal in tumors (*n* = 4) of mice treated with MSCs (**F**) and OAd-MSC (**G**). Unpaired *t* test. **, *P* < 0.01. **H,***Ex vivo* quantification of fluorescence signal in organs of mice treated with OAd-MSC. Data are represented as mean + SD.

As we hypothesize that a silent delivery of the cell vehicle would increase its migration to the malignancy, we investigated the peripheral immune response after administration of the treatment and its involvement in the homing to the tumor site. We then performed new *in vivo* experiments in our mouse model to elucidate the mechanism of action underlying the improved antitumor effect of OAd-MSC TLR4^−/−^ (Fig. 4).

Immunocompetent mice bearing luciferase transduced CMT64 tumors were treated with 1 × 10^5^ OAd-MSC WT or OAd-MSC TLR4^−/−^ and blood samples were obtained at 48 hours to study their inflammatory status, hemogram, and biochemistry (Fig. 4A). In line with our hypothesis, complete blood count from mice treated with OAd-MSC WT showed significant increase of white blood cells, monocytes, neutrophils, and lymphocytes that was not observed after administration of OAd-MSC TLR4^−/−^ (Fig. 4B). Indeed, the response of OAd-MSC TLR4^−/−^ was similar to the induced by the noninfected MSCs (Supplementary Fig. S7A). In the same direction, this silent response was also recapitulated in the cytokine analysis from serum, as OAd-MSC TLR4^−/−^ induced lower proinflammatory profile in the peripheral blood than OAd-MSC WT (Fig. 4C).

We also performed a biochemistry analysis of blood samples to study the possible tissue toxicity of the treatment. However, no relevant differences between groups treated with PBS or any OAd-MSC were observed, which indeed accentuates the low toxicity of our cellular virotherapy (Supplementary Fig. S7B).

These results indicate that administration of silent cells (OAd-MSC TLR4^−/−^) induces lower systemic inflammatory response than administration of OAd-MSC WT.

### OAd-MSC TLR4^−/−^ Presents Higher Tumor Homing *In Vivo*

We then studied the tumor homing of the treatment *in vivo* by tracking the MSCs and OAd-MSC labeled with the fluorescent marker DIR prior to administration. A total of 48 hours after administration, OAd-MSC TLR4^−/−^ presented significant higher tumor homing than OAd-MSC WT (Fig. 4D–G). It is interesting to note that OAd-MSC TLR4^−/−^ showed similar tumor homing than noninfected MSCs WT and MSCs TLR4^−/−^, while homing of OAd-MSC WT to the tumor site was reduced (Fig. 4F). Biodistribution of the treatment to other organs was also studied in spleen, kidney, lungs, and liver, but no relevant differences were observed between OAd-MSC WT and OAd-MSC TLR4^−/−^ (Fig. 4E–H).

These results indicate that silent cells (OAd-MSC TLR4^−/−^) present higher *in vivo* migration capacity to the tumor site than OAd-MSC WT.

### OAd-MSC TLR4^−/−^ Induces the Activation of NFκB Pathway in the Tumor

We speculated that the higher migration of OAd-MSC TLR4^−/−^ to the tumor site could generate high local inflammation in the tumor microenvironment. This *in situ* inflammation would indeed explain the high tumor infiltration previously observed after treatment with OAd-MSC TLR4^−/−^ (Fig. 2) and the resulting enhanced antitumor effect.

**FIGURE 5:**
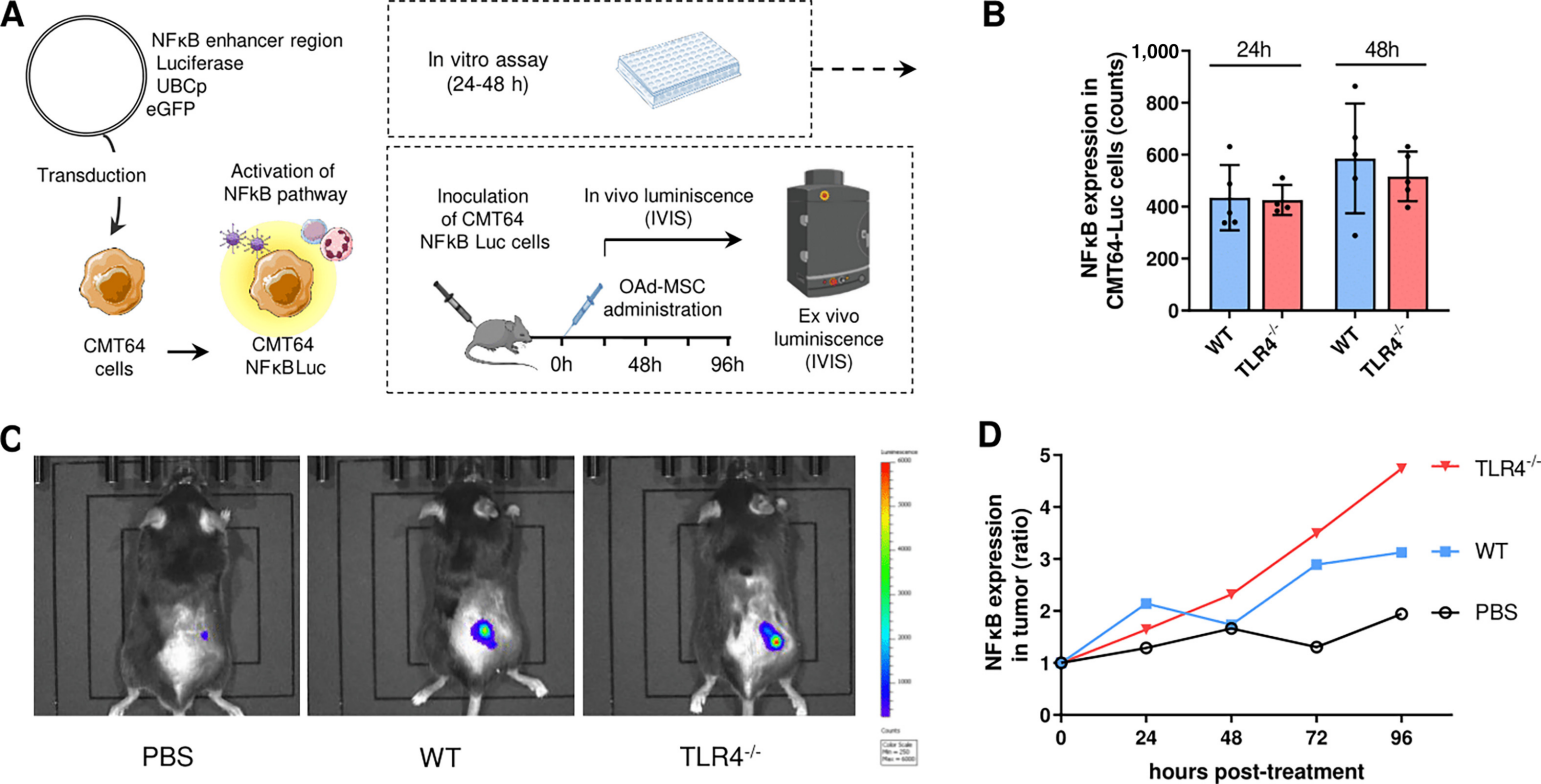
OAd-MSC TLR4^−/−^ induces the activation of NFκB in the tumor. **A,** Schematic illustration of experimental design. **B,***In vitro* expression of NFκB pathway detected by bioluminiscence in CMT64 NFκB-Luc tumor cells after coculture with OAd-MSC for 24 and 48 hours (*n* = 5). **C,***In vivo* expression of NFκB pathway in the tumor at endpoint after treatment with PBS, OAd-MSC WT and OAd-MSC TLR4^−/−^. All images are displayed at the same bioluminescence scale. **D,***In vivo* activation ratio of NFκB pathway in the tumor at different timepoints (*n* = 5).

To validate this mechanism of action, activation of NFκB pathway was studied in the tumor cells, as this route serves as a pivotal mediator of inflammatory response. Thus, CMT64 NFκB-Luc cells were generated using a luciferase reporter system (Fig. 5A). We first studied the *in vitro* expression of NFκB in tumor cells after coculture with OAd-MSC for 24 and 48 hours, but no differences were observed between OAd-MSC WT and OAd-MSC TLR4^−/−^ (Fig. 5B).

We also studied the *in vivo* proinflammatory response in the tumor. C57BL/6 immunocompetent mice bearing CMT64 NFκB-Luc tumors were treated with OAd-MSC and luminescence was measured for 4 days (Fig. 5A). Interestingly, although mice treated with OAd-MSC WT and OAd-MSC TLR4^−/−^ induced the activation of NFκB in the tumors, this expression tended to be higher in the tumors treated with OAd-MSC TLR4^−/−^ (Fig. 5C and D).

In overall, these results indicate that the better antitumor effect of OAd-MSC TLR4^−/−^ is highly related to the triggered response of the immune system. Indeed, OAd-MSC TLR4^−/−^ induced high proinflammatory response in the tumor microenvironment.

### OAd-MSC MyD88^−/−^ Confirms the *In Vitro* Mechanism of Action of Silent Cells Model

The study of OAd-MSC TLR4^−/−^ demonstrated that silent cells as carriers for the delivery of OAd improve the antitumor efficacy of the therapy by increasing tumor homing. However, this improved effect could be mediated by the specific absence of TLR4.

**FIGURE 6:**
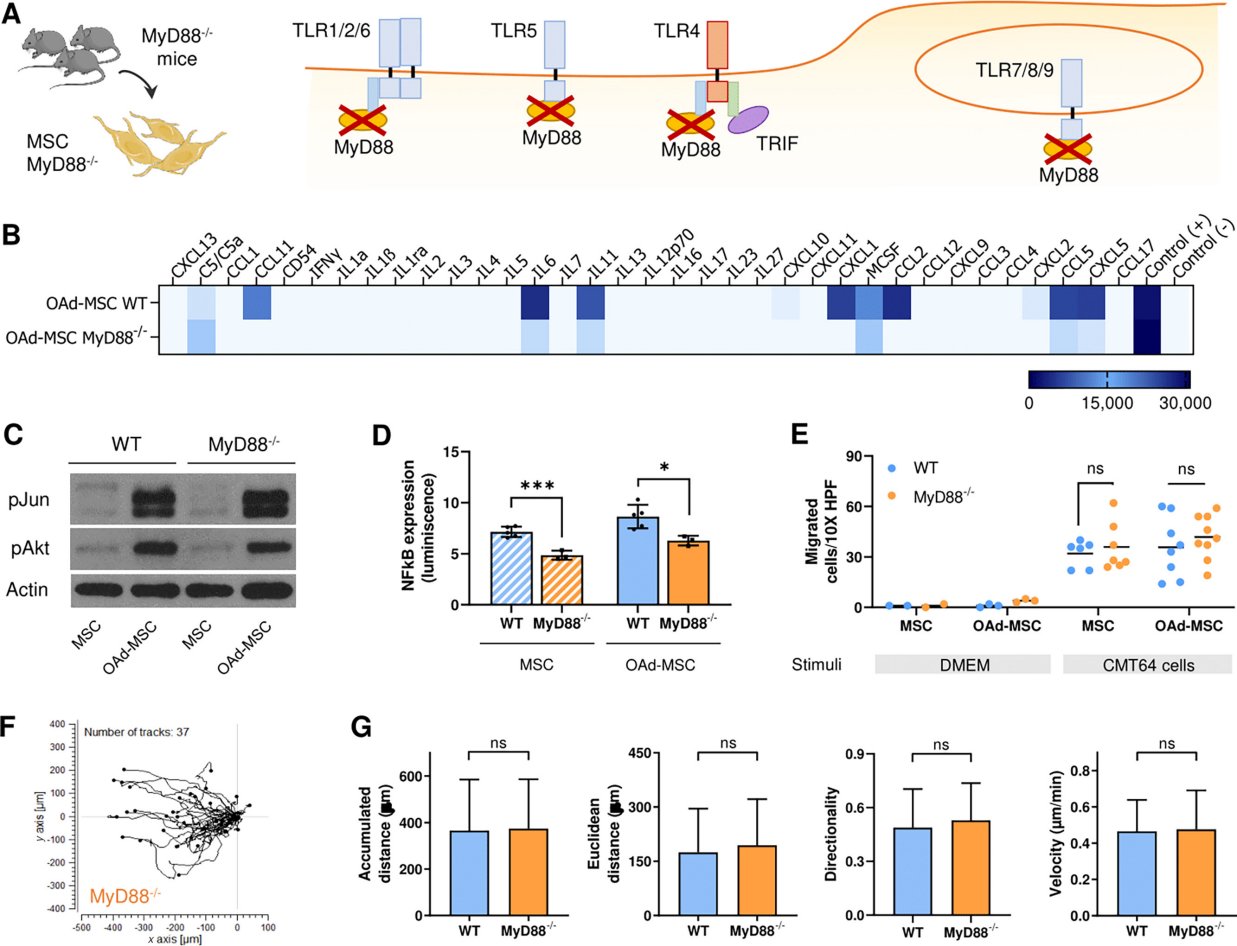
OAd-MSC MyD88^−/−^ presents similar signaling and *in vitro* migration than OAd-MSC TLR4^−/−^. **A,** Schematic illustration showing MyD88-dependent TLR signaling. **B,** Heatmap showing the inflammatory secretome at 24 hours after infection with OAd. Light blue represents the lowest expression while dark blue represents the highest expression. **C,** Protein expression of phospho-Jun (pJun), phospho-Akt (pAkt), and Actin analyzed by Western blot analysis at 24 hours. **D,** NFκB expression at 24 hours reported by luminescence. Unpaired *t* test. *, *P* < 0.05; ***, *P* < 0.001. **E,** Transwell migration assay of MSCs and OAd-MSC toward CMT64 tumor cells at 24 hours (*n* = 6–8). DMEM alone as stimuli was used as negative control (*n* = 2–3). Migrated cells were quantified in 10X HPF. Two-way ANOVA followed by Tukey multiple comparisons test. ns, not significant; ***, *P* < 0.001. **F,** Trajectory plot of tracked OAd-MSC MyD88^−/−^ (*n* = 37) toward CMT64 tumor cells. **G,** Parameters measured from tracked cell trajectories of migrating OAd-MSC MyD88^−/−^ toward tumor cells. For the whole figure, stripped bars correspond to mock-infected MSCs while solid bars correspond to OAd-MSC (WT cells in blue; MyD88^−/−^ cells in yellow).

To make the study extensive to the whole TLR signaling pathway, we used mouse MSCs knockout for MyD88 (MSCs MyD88^−/−^) as a wider model of silent cells. MyD88 is the canonical adaptor for inflammatory signaling pathways downstream of members of the TLR and IL1 receptor families. With the exception of TLR3, all TLRs—either intracellular or extracellular—initiate a MyD88-dependent signaling pathway (ref. 23; Fig. 6A). We then performed the same *in vitro* experiments using MSCs MyD88^−/−^ as an additional model of silent cells to validate our hypothesis.

OAd-infected MSCs MyD88^−/−^ (OAd-MSC MyD88^−/−^) also showed lower secretion of inflammatory cytokines than OAd-MSC WT (Fig. 6B). Although expression of total and phosphorylated Jun (pJun) and Akt (pAkt) was similar in OAd-MSC WT and OAd-MSC MyD88^−/−^ (Fig. 6C; Supplementary Fig. S8), NFκB expression was significantly lower in MyD88^−/−^ cells (MSCs or OAd-MSC) than in WT cells (Fig. 6D). *In vitro* migration of OAd-MSC WT or OAd-MSC MyD88^−/−^ toward CMT64 tumor cells showed no differences (Fig. 6E–G; Supplementary Video S3). In summary, these results are similar to those obtained using TLR4^−/−^ cells.

### OAd-MSC MyD88^−/−^ Confirms the *In Vivo* Mechanism of Action of Silent Cells Model

**FIGURE 7:**
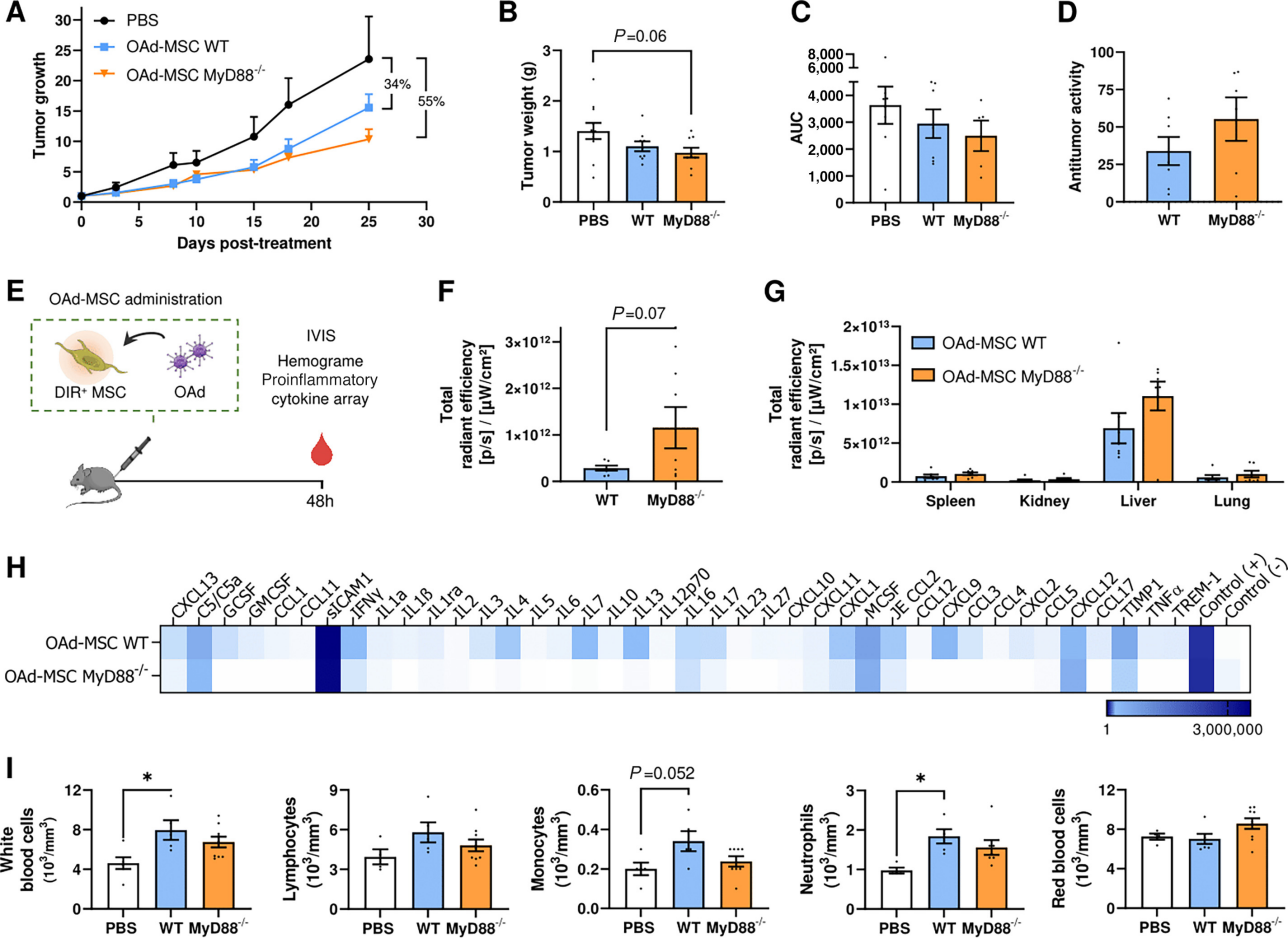
OAd-MSC MyD88^−/−^ also improves the antitumor efficacy of OAd-MSC WT *in vivo*. **A,** Follow-up of tumor growth in mice treated with PBS (black), OAd-MSC WT (blue), or OAd-MSC MyD88^−/−^ (yellow) represented as mean + SEM (*n* = 7). **B,** Tumor weight of treated mice at endpoint. One-way ANOVA followed by Tukey multiple comparisons tests. **C,** AUC of treated groups expressed as mean + SEM (*n* = 7). **D,** Antitumor activity of OAd-MSC WT and OAd-MSC MyD88^−/−^ in relation to control group (*n* = 7). **E,** Schematic illustration of *in vivo* experimental design. **F,***In vivo* homing of OAd-MSC to tumor site 48 hours after treatment administration, expressed as *ex vivo* quantification of fluorescence (DIR^+^) signal in tumors of mice treated with OAd-MSC (*n* = 7). Unpaired *t* test. **G,***In vivo* homing of OAd-MSC to different organs extracted at 48 hours, expressed as *ex vivo* quantification of fluorescence (DIR^+^) signal in tumors of treated mice. **H,** Heatmap showing inflammatory cytokines in serum at 48 hours after administration of OAd-MSC WT or OAd-MSC MyD88^−/−^ (pool from *n* = 4). White represents the lowest expression while dark blue represents the highest expression. **I,** Immune populations from complete blood count at 48 hours after treatment administration (*n* = 5–7). One-way ANOVA followed by Tukey multiple comparisons test. *, *P* < 0.05.

The antitumor efficacy of MyD88^−/−^ cells was also studied *in vivo*. Immunocompetent C57BL/6 mice bearing CMT64 tumors were treated with OAd-MSC MyD88^−/−^ (1 × 10^5^ cells/mouse) every 5 days. Interestingly, treatment with OAd-MSC MyD88^−/−^ also resulted in higher antitumor effect than OAd-MSC WT (Fig. 7A–D), showing similar results to the obtained after treatment with OAd-MSC TLR4^−/−^.

As we have previously demonstrated that the immune response after systemic administration and tumor homing of the treatment play key roles in the improved antitumor effect, we also studied these parameters after treatment with OAd-MSC MyD88^−/−^ (Fig. 7E). Indeed, OAd-MSC MyD88^−/−^ also showed higher tumor homing than OAd-MSC WT (Fig. 7F), while no remarkable differences were observed in the biodistribution to other organs (Fig. 7G). In line with the results obtained with OAd-MSC TLR4^−/−^, serum from mice treated with OAd-MSC MyD88^−/−^ showed lower levels of proinflammatory cytokines than those from mice treated with OAd-MSC WT (Fig. 7H). A similar tendency was also observed in different immune populations from the complete blood count (Fig. 7I).

In addition, our hypothesis was confirmed using MSC knockout for TLR9, an intracellular TLR that has been described to contribute to the innate immune response to adenoviral vectors (24). *In vivo* study showed that OAd-MSC TLR9^−/−^ presented similar antitumor effect than OAd-MSC TLR4^−/−^ and OAd-MSC MyD88^−/−^ (Supplementary Fig. S9).

These results show that OAd-MSC using silent cells increases the antitumor efficacy of OAd-MSC using WT cells, following a similar mechanism of action.

## Discussion

**FIGURE 8:**
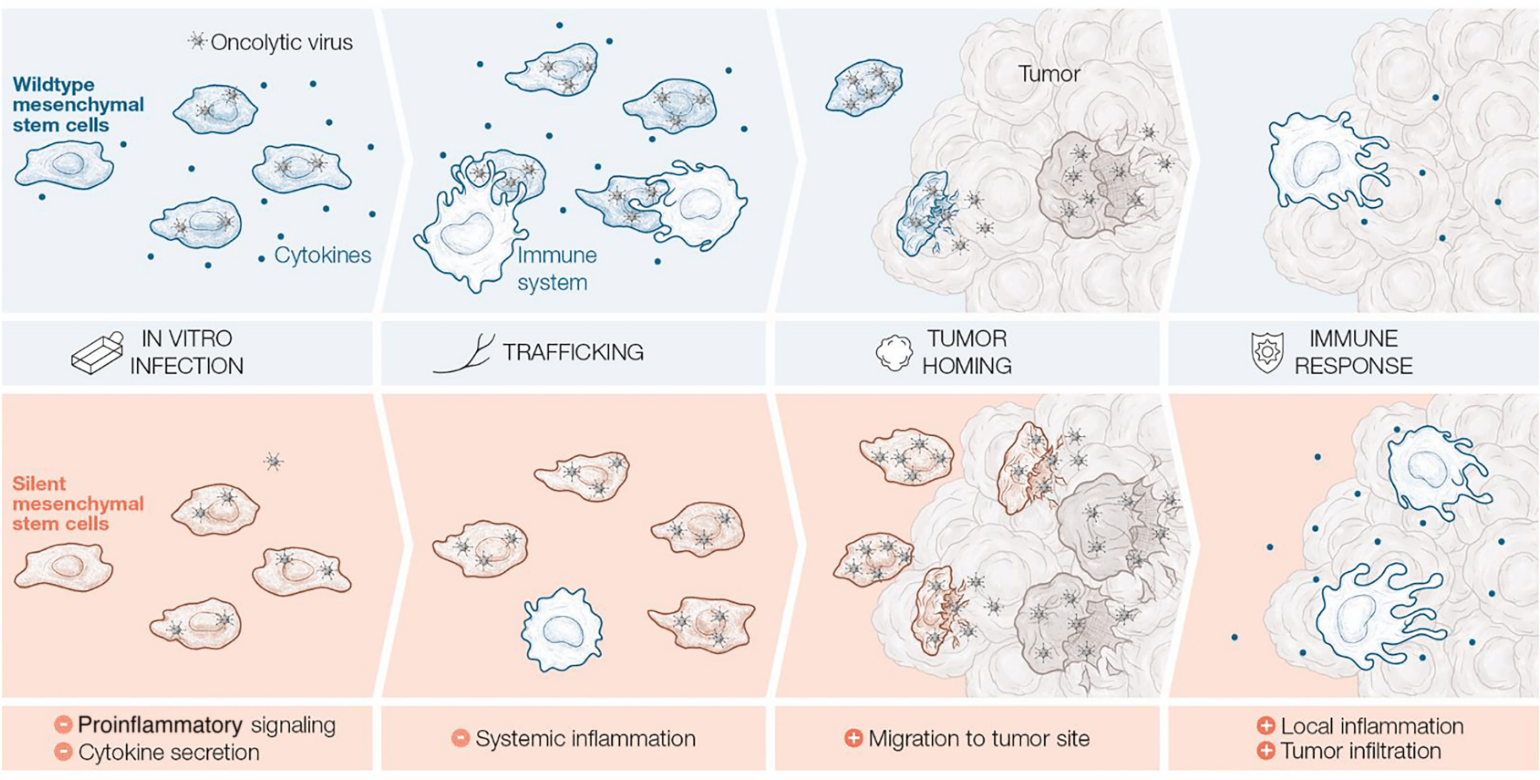
Mechanism of action of OAd-MSC. The use of silent cells presenting low proinflammatory signaling (down) would induce lower systemic inflammation upon systemic administration. This permissiveness would lead to increased tumor homing, thus inducing local inflammation and higher tumor infiltration of immune cells. Consistent with our hypothesis, the proposed mechanism of action underlies that therapies based on silent cells would result in better antitumor response.

Our study demonstrates that therapies based on cells presenting low proinflammatory profiles (silent cells) result in improved antitumor responses. Figure 8 shows the proposed mechanism of action. Briefly, the use of silent cells does not induce systemic inflammation in peripheral blood after administration. This absence of response leads to increased tumor homing, thus inducing local inflammation in the tumor and higher immune infiltration. As a result, increased antitumor efficacy is obtained.

In cancer immunotherapy, different agents are used to activate or boost the activation of the immune system to attack cancer cells. Thus, a key goal of immunotherapies is to establish inflammation in the tumor microenvironment, promoting innate immunity, trafficking and priming of T lymphocytes. In this regard, pattern recognition receptors (PRR) are crucial for initiating innate and adaptive immune responses, with activation of receptors like TLRs in the tumor microenvironment contributing to anticancer immunity. Following this idea, intratumoral immunotherapies using PRR agonists such as TLRs ligands are being explored to induce or enhance local inflammation and immune activation in the tumor (25, 26).

On the other hand, immunotherapies using mAbs or adoptive cell transfer are usually infused intravenously. However, these systemic agents often cause systemic inflammation and autoimmune-like reactions that may lead to on-target off-tumor toxicity (25, 27, 28). As a result, the effect of inflammation in cancer immunotherapy is two-edged and, although targeting inflammation is an important way for improving anticancer treatment, spatiotemporality context of this immune activation is of vital importance. For this reason, exploring therapeutic options that control modulation of the immune system with low systemic toxicity and high antitumor efficacy remains a challenge in cancer immunotherapies (29).

Here we confirm that cell-based therapies that induce low systemic inflammation after parenteral administration present higher tumor homing and, therefore, enhance the antitumor efficacy of the treatment. The cell-based therapy model used in the study comprehends MSCs carrying the OAd ICOVIR-5 (13), an immunotherapy called Celyvir in which our group has been working for more than 15 years (30). The clinical use of these treatment has already been demonstrated in preclinical studies (17, 18, 31–34), a veterinary trial of dogs presenting spontaneous solid tumors (35), and clinical trials of children presenting refractory solid tumors (EudraCT: 2008-000364-16, 2019-001154-26, 2020-004838-37) that resulted in clinical benefits—including complete remissions. More importantly, the systemic administration of this cell-based therapy showed an excellent safety profile (36–40).

A central goal in the development of cancer cell-based therapies is to enable targeted and controlled release of active substances in the desired organs or tissues (10). For this purpose, clearance of the inoculated treatment is particularly sensitive in the case of systemically delivered immunotherapies, especially virus-based therapies and those involving cell carriers or nanoparticles (5, 8, 41). Recently, it has been reviewed that IL6, TNFα, and IL1β play a central role in the toxicities of cancer immunotherapies (28). The good tolerance of our treatment may be explained by the low levels of these cytokines found in the serum after administration. Nevertheless, the systemic administration of this cell-based therapy using silent cells did show a milder profile of systemic inflammation—including lower secretion of proinflammatory cytokines—which shows even greater tolerance. Over the years, data from colleagues and our group pointed out the importance of the systemic inflammation and the intracellular signaling of the cell carrier in the antitumor efficacy. In this regard, Melen and colleagues showed that systemically delivered cells presenting a silent profile appear to achieve better clinical responses for the treatment of solid tumors (37). We indeed found that the low dose of our cell-based therapy showed better antitumor results than the high dose. Although it may be surprising, results from clinical trials also showed good clinical outcomes in patients treated with lower doses of Celyvir (37–39), which may be related to the lower systemic inflammation triggered by the reduced dose.

Following this idea, Rodríguez-Milla and colleagues also proposed that the absence of activation of certain pathways in the administered cells may improve the efficacy of the cell-based therapy (22). Here, our results using silent cells—knockout for the TLR pathway—demonstrate this paradigm and provide a mechanism of action for this improved effect. The immune escape ability of these silent cells avoids the systemic inflammation and reduces their attack and clearance by the immune system. As a result, viability of these silent cells is improved, and homing to the tumor site is increased. A similar strategy has been applied to the delivery of biomimetic nanoparticles camouflaged in cancer cell membranes, which also exhibited the abilities of immune escape and tumor homing (41, 42).

Our study revealed that the inflammatory status of systemically administered products is critical in cell-based therapies for cancer treatment. The double-edged sword role of the immune system in cancer is then brought to the fore: while cancer immunotherapies generally aim to boost local immune responses in the tumor microenvironment, low systemic inflammation after systemic administration of the treatment may indeed enhance their tumor homing and improve the overall antitumor effect. Finally, preselection of suitable cells would be key to produce effective clinical outcomes; in particular, the use of silent cells as cell carrier for oncolytic viruses increased tumor homing and enhances the antitumor effect of the therapy.

## Supplementary Material

Supplementary Figure S1 Validation of MSCs TLR4−/−

Supplementary Figure S2 High dose OAd-MSC TLR4−/− improves the antitumor efficacy of high dose OAd-MSC WT in vivo

Supplementary Figure S3 MSCs TLR4−/− do not induce any antitumor effect in vivo by itself

Supplementary Figure S4 OAd-MSC TLR4−/− induces changes in tumor-infiltration of innate and adaptive immune cells

Supplementary Figure S5 OAd-MSC WT and OAd-MSC TLR4−/− induce similar changes in immune populations of the spleen

Supplementary Figure S6 OAd-MSC WT and OAd-MSC TLR4−/− show similar expression of cell membrane receptors in vitro

Supplementary Figure S7 OAd-MSC TLR4−/− induces lower systemic pro-inflammatory response than OAd-MSC WT in vivo

Supplementary Figure S8 In vitro signaling of OAd-MSC MyD88−/−

Supplementary Figure S9 Silent OAd-MSC using TLR4−/−, TLR9−/− or MyD88−/− cells presents similar antitumor efficacy in vivo

Supplementary Video SV1 OAd-MSC WT

Supplementary Video SV2 OAd-MSC TLR4−/−

Supplementary Video SV3 OAd-MSC MyD88−/−
