# Supplementary material for: Toll-like Receptor Signaling–deficient Cells Enhance Antitumor Activity of Cell-based Immunotherapy by Increasing Tumor Homing
Source: Cancer Res Commun. 2023 Mar 1;3(3):347–60. doi: 10.1158/2767-9764.CRC-22-0365 (PMC9976589; doi:10.1158/2767-9764.CRC-22-0365)
Supplement: Supplementary Figure S1 — Validation of MSCs TLR4−/− [file crc-22-0365-s01.pdf]

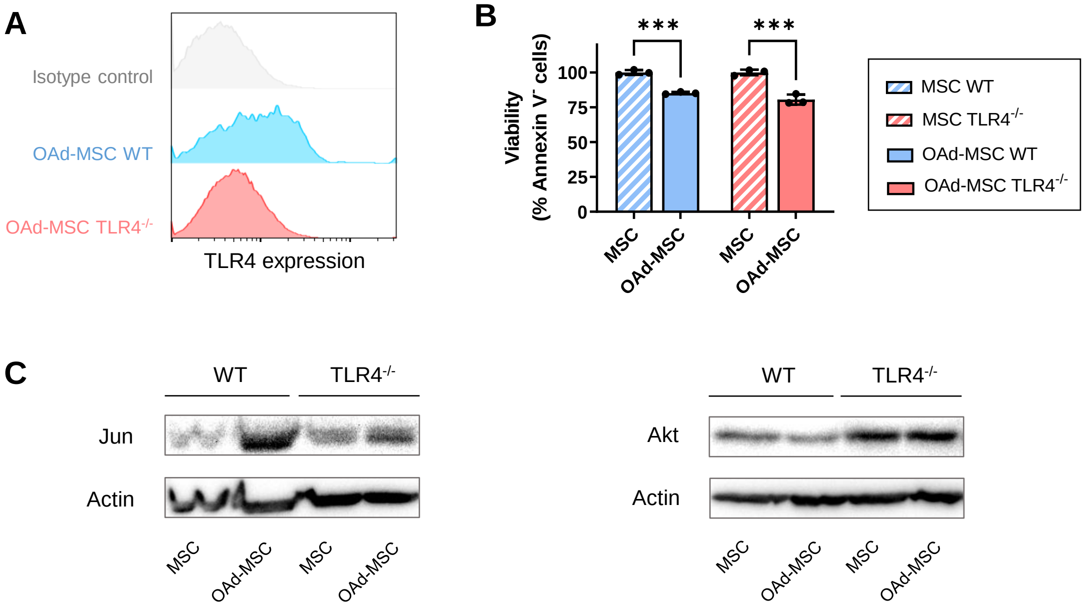

Supplementary Figure S1. Validation of MSCs TLR4$^{-/-}$. A, Confirmation of expression and absence of TLR4 in OAd-MSC WT and OAd-MSC TLR4$^{-/-}$, respectively, by flow cytometry. **B,** Viability of MSCs at 48 h after infection with the OAd ICOVIR-5 (OAd-MSC), measured with Annexin V apoptosis detection kit. Two-way followed by Tukey's multiple comparisons test. ***p < 0.001. For the whole figure, stripped bars correspond to mock-infected MSCs while solid bars correspond to OAd-MSCs (WT cells in blue; TLR4$^{-/-}$ cells in red). **C,** Protein expression of total Jun, Akt and corresponding Actin analyzed by WB at 24 h.
