# Supplementary material for: Toll-like Receptor Signaling–deficient Cells Enhance Antitumor Activity of Cell-based Immunotherapy by Increasing Tumor Homing
Source: Cancer Res Commun. 2023 Mar 1;3(3):347–60. doi: 10.1158/2767-9764.CRC-22-0365 (PMC9976589; doi:10.1158/2767-9764.CRC-22-0365)
Supplement: Supplementary Figure S2 — High dose OAd-MSC TLR4−/− improves the antitumor efficacy of high dose OAd-MSC WT in vivo [file crc-22-0365-s02.pdf]

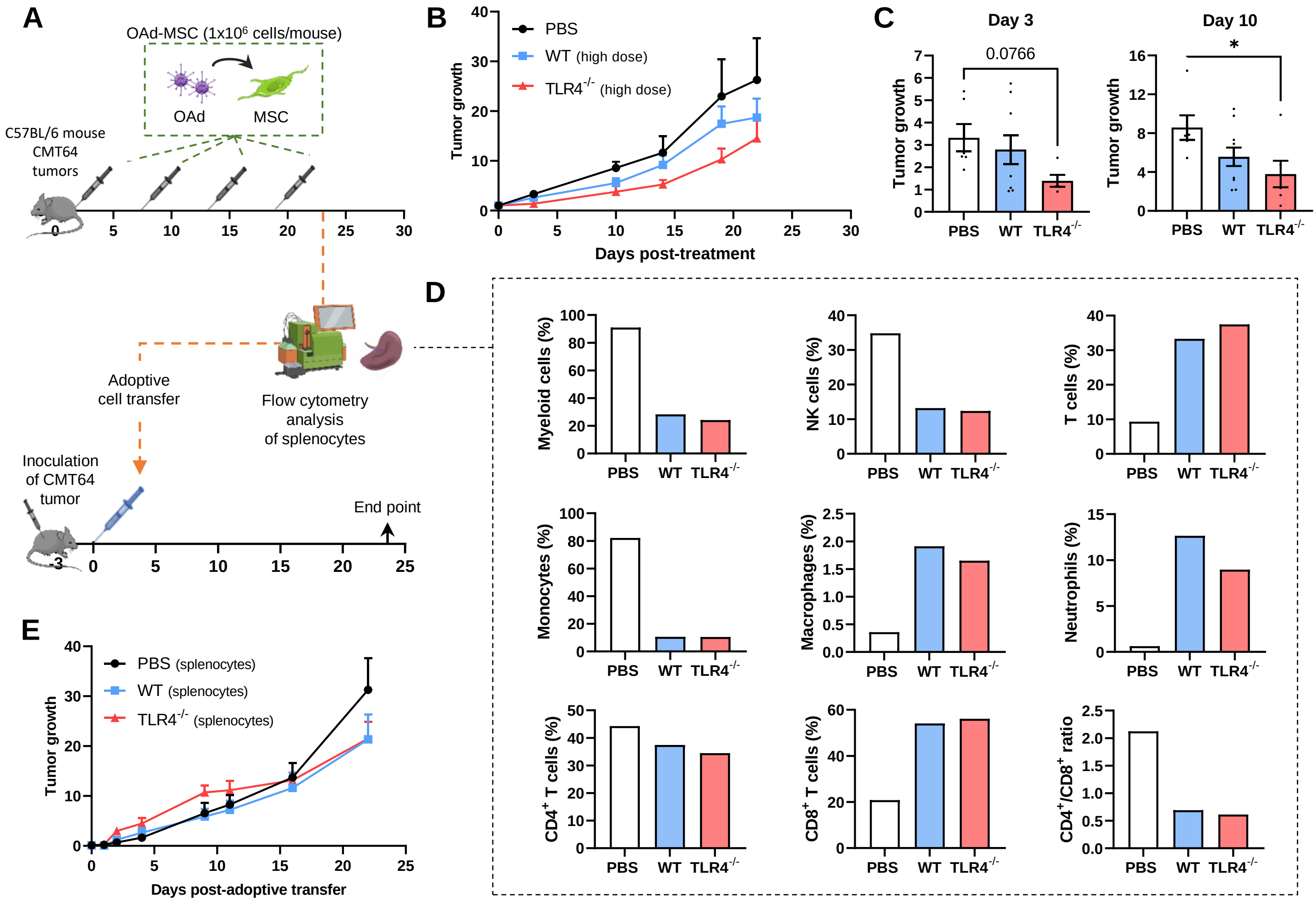

Supplementary Figure S2. High dose OAd-MSC TLR4$^{-/-}$ improves the antitumor efficacy of high dose OAd-MSC WT in vivo. A, Schematic illustration of in vivo experimental design for antitumor efficacy and adoptive cell transfer experiments. **B,** Follow-up of tumor growth in mice treated with PBS (black), high dose OAd-MSC WT (blue) or high dose OAd-MSC TLR4$^{-/-}$ (red) represented as mean + SEM (*n* = 6-10). **C,** Days showing statistical differences in tumor growth. One-way ANOVA followed by Tukey's multiple comparisons tests. *p < 0.05. **D,** Flow cytometry analysis of immune populations in spleens obtained at end point from treated mice represented as mean (pools from *n* = 5). **E,** Follow-up of tumor growth after adoptive transfer of splenocytes from previous OAd-MSC-treated groups, represented as mean + SEM (*n* = 5).
