# Supplementary material for: Toll-like Receptor Signaling–deficient Cells Enhance Antitumor Activity of Cell-based Immunotherapy by Increasing Tumor Homing
Source: Cancer Res Commun. 2023 Mar 1;3(3):347–60. doi: 10.1158/2767-9764.CRC-22-0365 (PMC9976589; doi:10.1158/2767-9764.CRC-22-0365)
Supplement: Supplementary Figure S3 — MSCs TLR4−/− do not induce any antitumor effect in vivo by itself [file crc-22-0365-s03.pdf]

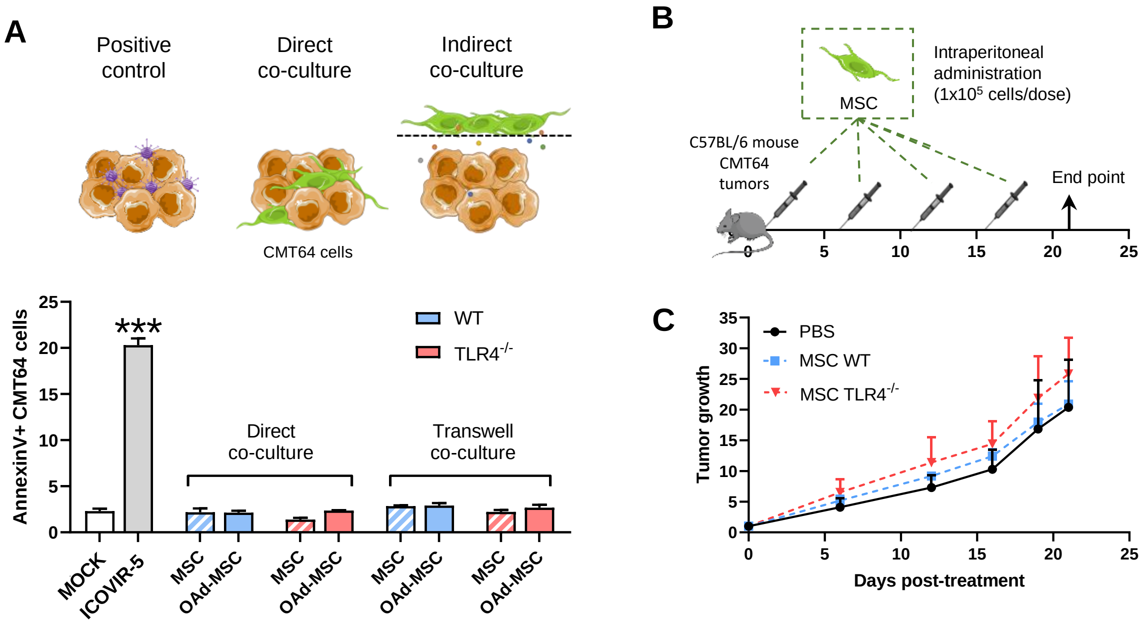

Supplementary Figure S3. MSCs TLR4$^{-/-}$ do not induce any antitumor effect in vivo by itself. A, Cytotoxicity in CMT64 tumor cells after direct and transwell co-cultures with MSCs and OAd-MSC. The experiment was performed at short time (48 h) in order to avoid the antitumor effect of the released virus. MOCK-infected CMT64 cells were used as negative control (white), while CMT64 cells infected with the OAd ICOVIR-5 were used as positive control (grey). Statistical analysis refers to negative control. Two-way followed by Tukey's multiple comparisons test. ***p < 0.001. **B,** Schematic illustration of in vivo experimental design. **C,** Follow-up of tumor growth in mice treated with PBS (black), MSCs WT (dotted blue line) or MSCs TLR4$^{-/-}$ (dotted red line) represented as mean + SEM and tumor growth at end point (n = 5).
