# Supplementary material for: Toll-like Receptor Signaling–deficient Cells Enhance Antitumor Activity of Cell-based Immunotherapy by Increasing Tumor Homing
Source: Cancer Res Commun. 2023 Mar 1;3(3):347–60. doi: 10.1158/2767-9764.CRC-22-0365 (PMC9976589; doi:10.1158/2767-9764.CRC-22-0365)
Supplement: Supplementary Figure S4 — OAd-MSC TLR4−/− induces changes in tumor-infiltration of innate and adaptive immune cells [file crc-22-0365-s04.pdf]

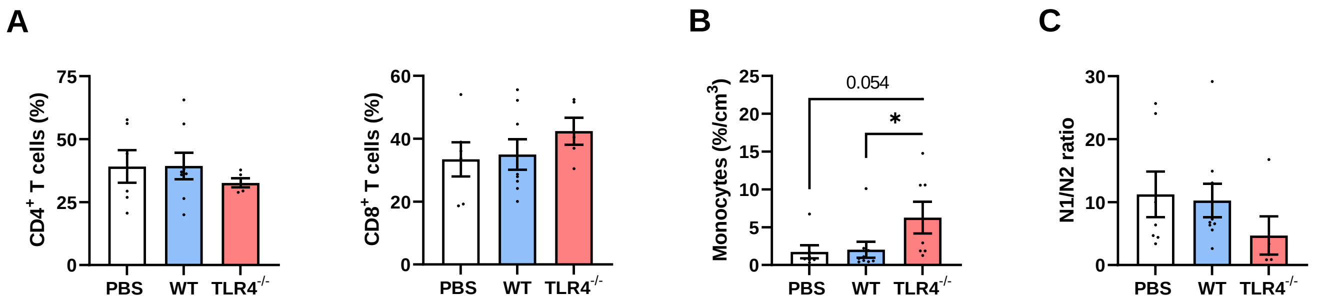

Supplementary Figure S4. OAd-MSC TLR4$^{-/-}$ induces changes in tumor-infiltration of innate and adaptive immune cells. A, Percentage of CD4^{+} and CD8^{+} T cell subsets from tumor-infiltrating lymphocytes. **B,** Density of tumor-infiltrating monocytes at end point (24 days after first treatment administration), expressed as percentage per cm^{3} of tumor. **C,** Ratio of pro-inflammatory/anti-inflammatory status of neutrophils (N1/N2 ratio). n = 5-9. One-way ANOVA followed by Tukey's multiple comparisons test. *$p < 0.05$.
