# Supplementary material for: Toll-like Receptor Signaling–deficient Cells Enhance Antitumor Activity of Cell-based Immunotherapy by Increasing Tumor Homing
Source: Cancer Res Commun. 2023 Mar 1;3(3):347–60. doi: 10.1158/2767-9764.CRC-22-0365 (PMC9976589; doi:10.1158/2767-9764.CRC-22-0365)
Supplement: Supplementary Figure S5 — OAd-MSC WT and OAd-MSC TLR4−/− induce similar changes in immune populations of the spleen [file crc-22-0365-s05.pdf]

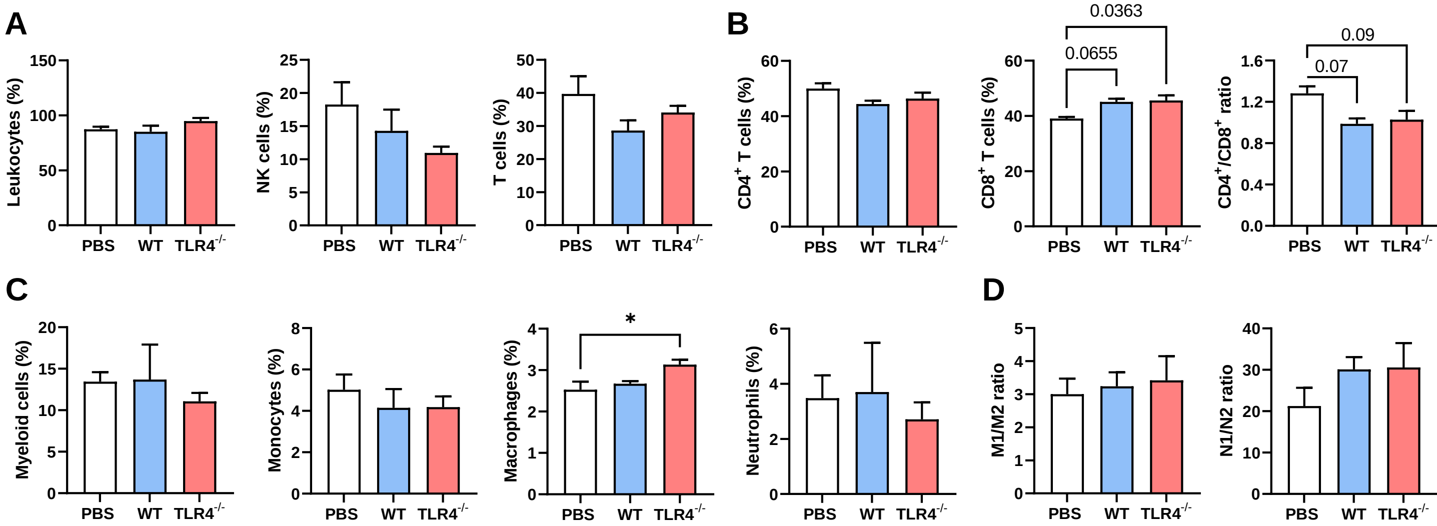

Supplementary Figure S5. OAd-MSC WT and OAd-MSC TLR4$^{-/-}$ induce similar changes in immune populations of the spleen. A, Percentage of leukocytes, NK cells and T cells in spleen (*n* = 3-4). **B,** Percentage of CD4^{+} and CD8^{+} subsets from T cells, as well as the CD4^{+}/CD8^{+} ratio. **C,** Percentage of innate immune populations in the spleen. **D,** Ratio of pro-inflammatory/anti-inflammatory status of macrophages (M1/M2 ratio) and neutrophils (N1/N2). One-way ANOVA followed by Tukey's multiple comparisons tests. *$p < 0.05$.
