# Supplementary material for: Toll-like Receptor Signaling–deficient Cells Enhance Antitumor Activity of Cell-based Immunotherapy by Increasing Tumor Homing
Source: Cancer Res Commun. 2023 Mar 1;3(3):347–60. doi: 10.1158/2767-9764.CRC-22-0365 (PMC9976589; doi:10.1158/2767-9764.CRC-22-0365)
Supplement: Supplementary Figure S6 — OAd-MSC WT and OAd-MSC TLR4−/− show similar expression of cell membrane receptors in vitro [file crc-22-0365-s06.pdf]

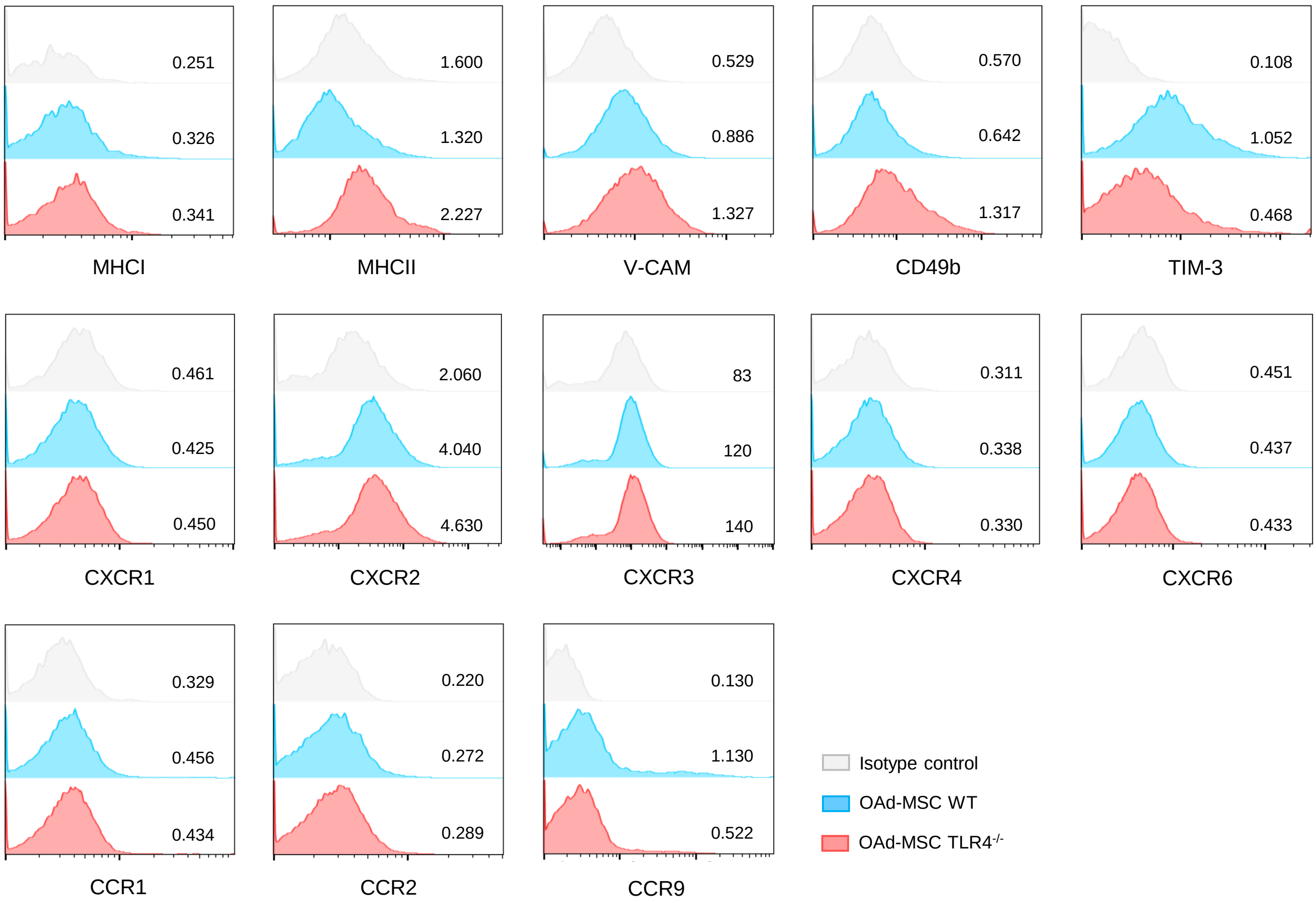

Supplementary Figure S6. OAd-MSC WT and OAd-MSC TLR4$^{-/-}$ show similar expression of cell membrane receptors in vitro. Representative histograms of flow cytometry analysis of OAd-MSC WT (blue) and OAd-MSC TLR4$^{-/-}$ (red) at 24 h. Mean fluorescence intensity (MFI) is indicated for each histogram.
