# Supplementary material for: Toll-like Receptor Signaling–deficient Cells Enhance Antitumor Activity of Cell-based Immunotherapy by Increasing Tumor Homing
Source: Cancer Res Commun. 2023 Mar 1;3(3):347–60. doi: 10.1158/2767-9764.CRC-22-0365 (PMC9976589; doi:10.1158/2767-9764.CRC-22-0365)
Supplement: Supplementary Figure S7 — OAd-MSC TLR4−/− induces lower systemic pro-inflammatory response than OAd-MSC WT in vivo [file crc-22-0365-s07.pdf]

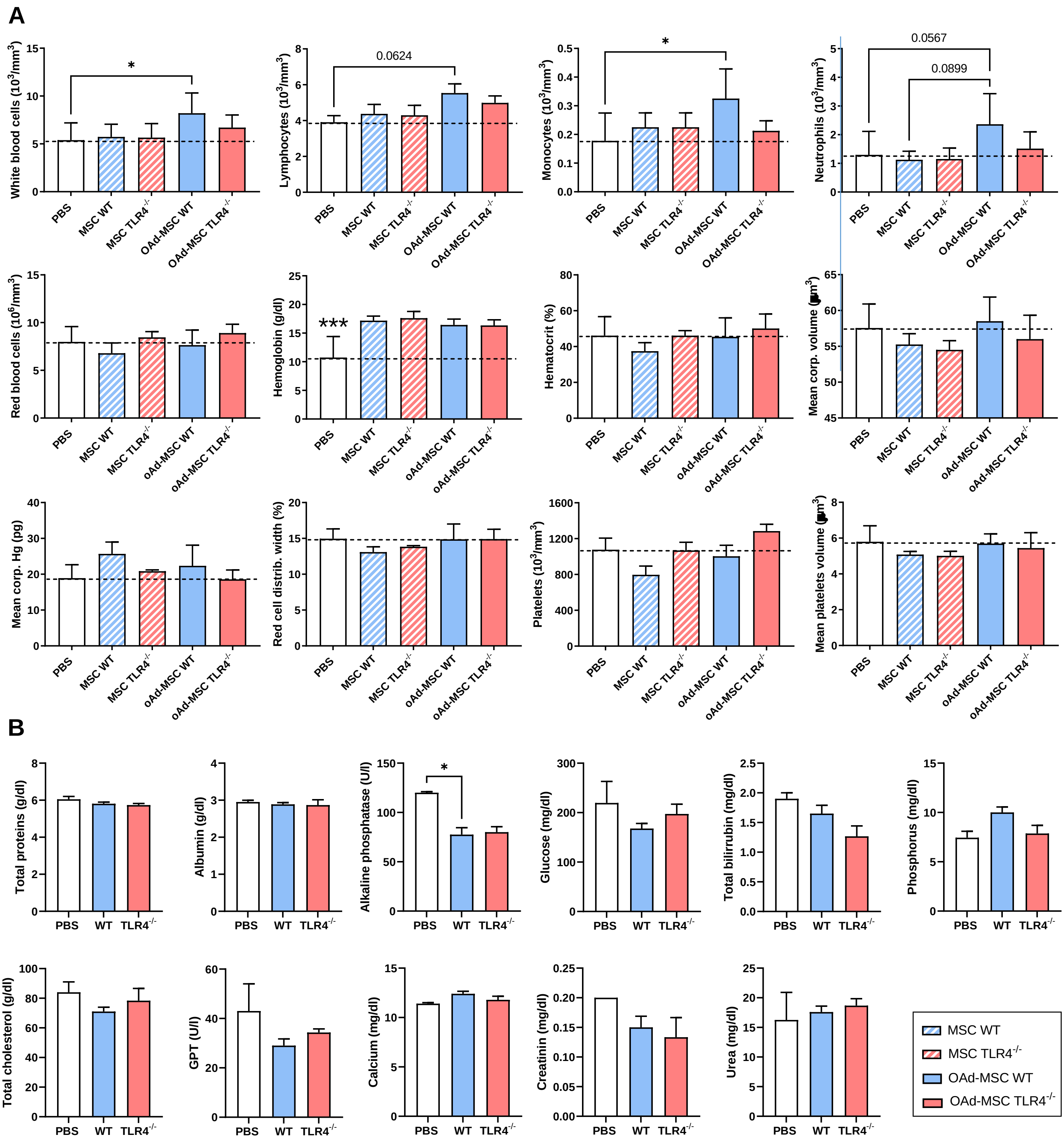

Supplementary Figure S7. OAd-MSC TLR4$^{-/-}$ induces lower systemic pro-inflammatory response than OAd-MSC WT in vivo. A, Immune populations and parameters from complete blood count obtained at 48 h after administration of PBS (white, *n* = 9), MSCs WT (blue with white stripes, *n* = 4), MSCs TLR4$^{-/-}$ (red with white stripes, *n* = 4), OAd-MSC WT (blue, *n* = 8) and OAd-MSC TLR4$^{-/-}$ (red, *n* = 8). Dotted lines indicate the mean of PBS group for each parameter. **B,** Biochemistry analysis from blood obtained at 48 h after administration of PBS (white), OAd-MSC WT (blue) and OAd-MSC TLR4$^{-/-}$ (red); *n* = 3-8. One-way ANOVA followed by Tukey's multiple comparisons tests. *p < 0.05.
