# Supplementary material for: Toll-like Receptor Signaling–deficient Cells Enhance Antitumor Activity of Cell-based Immunotherapy by Increasing Tumor Homing
Source: Cancer Res Commun. 2023 Mar 1;3(3):347–60. doi: 10.1158/2767-9764.CRC-22-0365 (PMC9976589; doi:10.1158/2767-9764.CRC-22-0365)
Supplement: Supplementary Figure S8 — In vitro signaling of OAd-MSC MyD88−/− [file crc-22-0365-s08.pdf]

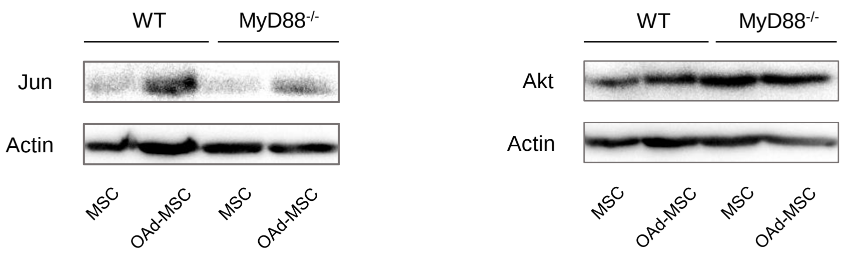


**Supplementary Figure S8. In vitro signaling of OAd-MSC MyD88$^{-/-}$.** Protein expression of total Jun, Akt and corresponding Actin analyzed by WB at 24 h.
