# Supplementary material for: Toll-like Receptor Signaling–deficient Cells Enhance Antitumor Activity of Cell-based Immunotherapy by Increasing Tumor Homing
Source: Cancer Res Commun. 2023 Mar 1;3(3):347–60. doi: 10.1158/2767-9764.CRC-22-0365 (PMC9976589; doi:10.1158/2767-9764.CRC-22-0365)
Supplement: Supplementary Figure S9 — Silent OAd-MSC using TLR4−/−, TLR9−/− or MyD88−/− cells presents similar antitumor efficacy in vivo [file crc-22-0365-s09.pdf]

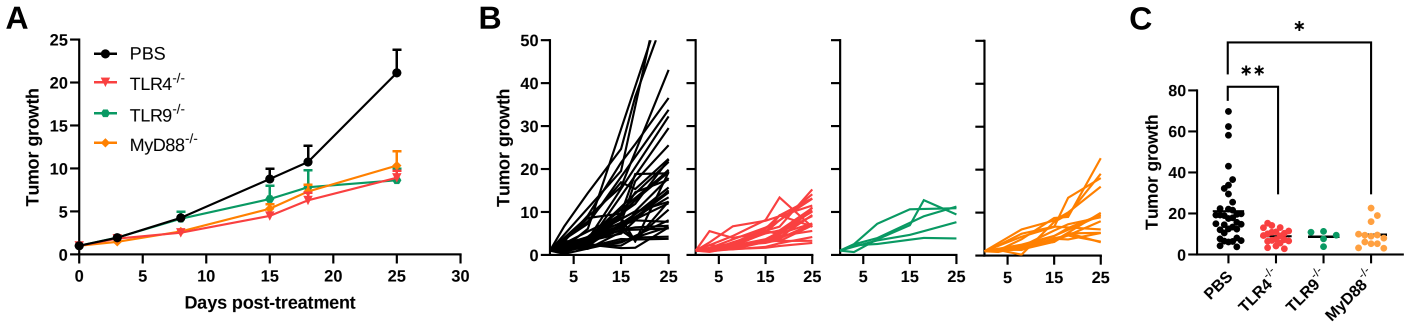

Supplementary Figure S9. Silent OAd-MSC using TLR4$^{-/-}$, TLR9$^{-/-}$ or MyD88$^{-/-}$ cells presents similar antitumor efficacy in vivo. A, B, Follow-up of tumor growth in mice treated with PBS (black, *n* = 36), OAd-MSC TLR4$^{-/-}$ (red, *n* = 20), OAd-MSC TLR9$^{-/-}$ (green, *n* = 4) or OAd-MSC MyD88$^{-/-}$ (yellow, *n* = 14) represented as (A) mean + SEM and (B) individual values. **C,** Tumor growth of mice treated with PBS, OAd-MSC TLR4$^{-/-}$, OAd-MSC TLR9$^{-/-}$ or OAd-MSC MyD88$^{-/-}$ at end point. One-way ANOVA followed by Tukey's multiple comparisons tests. *p < 0.05, **p < 0.01, ***p < 0.001.
